# The birth of a human-specific neural gene by incomplete duplication and gene fusion

**DOI:** 10.1186/s13059-017-1163-9

**Published:** 2017-03-09

**Authors:** Max L. Dougherty, Xander Nuttle, Osnat Penn, Bradley J. Nelson, John Huddleston, Carl Baker, Lana Harshman, Michael H. Duyzend, Mario Ventura, Francesca Antonacci, Richard Sandstrom, Megan Y. Dennis, Evan E. Eichler

**Affiliations:** 1 Department of Genome Sciences, University of Washington School of Medicine, 3720 15 Ave NE, S413C, Box 355065, Seattle, WA 98195-5065 USA; 2 Howard Hughes Medical Institute, University of Washington, Seattle, WA 98195 USA; 3 Department of Biology, University of Bari, Bari, 70121 Italy; 4 Altius Institute for Biomedical Sciences, Seattle, WA 98121 USA; 5 Genome Center, MIND Institute, and Department of Biochemistry & Molecular Medicine, University of California, Davis, 95616 CA USA

**Keywords:** Evolution, Segmental duplication, Duplicate genes, Gene fusion, Long-read sequencing, 1q21 microdeletion/microduplication syndrome

## Abstract

**Background:**

Gene innovation by duplication is a fundamental evolutionary process but is difficult to study in humans due to the large size, high sequence identity, and mosaic nature of segmental duplication blocks. The human-specific gene hydrocephalus-inducing 2, *HYDIN2*, was generated by a 364 kbp duplication of 79 internal exons of the large ciliary gene *HYDIN* from chromosome 16q22.2 to chromosome 1q21.1. Because the *HYDIN2* locus lacks the ancestral promoter and seven terminal exons of the progenitor gene, we sought to characterize transcription at this locus by coupling reverse transcription polymerase chain reaction and long-read sequencing.

**Results:**

5' RACE indicates a transcription start site for *HYDIN2* outside of the duplication and we observe fusion transcripts spanning both the 5' and 3' breakpoints. We observe extensive splicing diversity leading to the formation of altered open reading frames (ORFs) that appear to be under relaxed selection. We show that *HYDIN2* adopted a new promoter that drives an altered pattern of expression, with highest levels in neural tissues. We estimate that the *HYDIN* duplication occurred ~3.2 million years ago and find that it is nearly fixed (99.9%) for diploid copy number in contemporary humans. Examination of 73 chromosome 1q21 rearrangement patients reveals that *HYDIN2* is deleted or duplicated in most cases.

**Conclusions:**

Together, these data support a model of rapid gene innovation by fusion of incomplete segmental duplications, altered tissue expression, and potential subfunctionalization or neofunctionalization of *HYDIN2* early in the evolution of the *Homo* lineage.

**Electronic supplementary material:**

The online version of this article (doi:10.1186/s13059-017-1163-9) contains supplementary material, which is available to authorized users.

## Background

Gene duplication has long been hypothesized to be an important source of evolutionary innovation [1]. The great ape lineage that includes humans has experienced a surge of interspersed segmental duplications over the last 10–15 million years [2, 3]. While large, highly identical duplications sensitize the human genome to recurrent rearrangements associated with disease [4–7], they also have the potential to drive the emergence of novel duplicate genes and functions [8]. The identification of functional duplicate genes, however, is difficult, as these duplications are typically large, highly identical, and clustered into complex mosaic structures juxtaposing sequence blocks of diverse origin [9]. Furthermore, duplicated regions of the genome are frequently misassembled and are the source of extensive copy number variation in human populations [10, 11].

Considerable attention has been focused on the identification of duplicate genes that have emerged since the human–chimpanzee divergence because of their potential to contribute to human-specific traits [6, 12, 13]. Already two such genes, *SRGAP2C* and *ARHGAP11B*, have been functionally characterized by heterologous expression studies in mouse suggesting potential roles of the duplicates in increasing dendritic spine density [14] and expanding the number of cortical neurons [15], respectively. Both duplicate genes are nearly fixed for copy number in human populations but absent from all non-human primates [15–18]. In addition, both genes carry only a subset of the exons of the ancestral gene due to incomplete segmental duplication of the progenitor locus, supporting the hypothesis that truncation may facilitate neofunctionalization [8, 16].

Chromosome 1q21.1 is one of the largest regions of human-specific segmental duplication blocks in the human genome [6, 19] and, as such, is a reservoir for human-specific transcripts and genes [13, 20]. The presence of large blocks of directly oriented, highly identical duplicated sequence renders this region genetically unstable [7]. Specifically, recurrent deletions and duplications occur at this locus and have been associated with cognitive and motor impairment, articulation abnormalities, and hypotonia [21–23]. Duplication carriers show an increased prevalence of autism spectrum disorder (ASD) and macrocephaly, while deletion carriers show an increased prevalence of microcephaly.

The human-specific gene *HYDIN2* was previously mapped to this region of chromosome 1q21 [24], but because it was contained within an assembly gap in GRCh37/hg19, its role in 1q21 rearrangement syndromes remained uncertain. It was postulated that *HYDIN2* might contribute to the reciprocal macrocephaly/microcephaly phenotype [22], because mutation of the ancestral *HYDIN* gene leads to hydrocephalus in mouse [25]. In humans, however, recessive mutations in *HYDIN* were found associated with primary ciliary dyskinesia without hydrocephalus [26]. As such, whether dosage of *HYDIN2* plays a role in the 1q21.1 microdeletion/microduplication phenotype has remained unanswered.

With the initial discovery of the *HYDIN* duplication [24], two features of the derived locus were noted. First, although the gene duplication was truncated and did not include the promoter, transcription was observed at *HYDIN2*. Second, *HYDIN2* transcripts appeared to derive primarily from neuronal sources, in contrast to the ciliated tissues from which *HYDIN* transcripts were originally cloned. We sought to investigate the origin and significance of *HYDIN2* transcription by reconstructing the evolutionary history of this locus, assessing patterns of human genetic variation in both normal and disease populations, and exploring transcript diversity in human tissues.

## Results

### Molecular evolution and breakpoint analyses

**Fig. 1:**
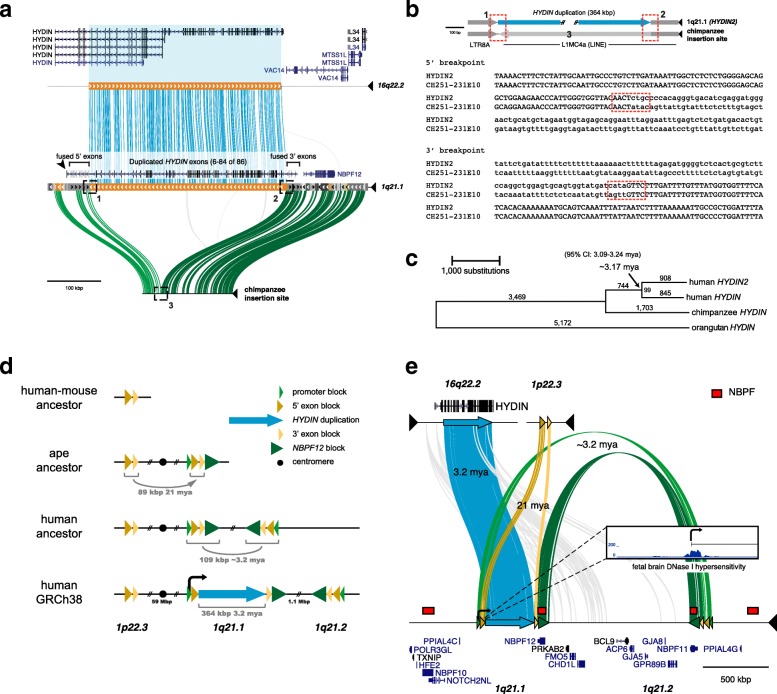
*HYDIN* duplication and evolution. **a** Comparison of human genomic sequence from the donor locus on chromosome 16q22.2 (*top*) to the acceptor locus on chromosome 1q21.1 (*middle*) shows a 364 kbp duplication (*blue lines*) including exons 6-84 of the 86-exon ancestral gene *HYDIN*, visualized using Miropeats [74]. Connecting lines indicate nearly identical segments (s = 1000). The orthologous insertion site (*bottom*) prior to insertion based on sequencing of chimpanzee BAC (CH251-231E10) is shown with homology indicated (*green lines*). Human gene annotation (GENCODE) as well as the location of the exons found in fusion transcripts, breakpoints (*dashed boxes*), and acquired novel promoter (*arrowhead*) are depicted. **b** Sequence alignment of the 5' and 3' breakpoints (*dashed red boxes*) shows that the duplication integrated at the boundary of an LTR and LINE repeat with the concomitant loss of 841 bp of LINE sequence based on analysis of the chimpanzee orthologous sequence. Uppercase bases are beyond the breakpoint while lowercase bases indicate a break in homology. Numbers are as indicated in (**a**). **c** A neighbor-joining phylogenetic tree based on a 315,349 bp MSA using the human paralogs as well as orthologous chimpanzee and orangutan sequences. Based on the genetic distance (Kimura 2-parameter) and assuming a human–chimpanzee divergence of 6 million years ago (mya), we estimate the duplication occurred ~3.17 mya (95% CI: 3.09–3.24 mya, bootstrap method). **d** The model depicts the simplified evolutionary history of the *HYDIN2* genomic locus as a series of juxtaposed segmental duplications that contributed novel exons and regulatory machinery. Human-mouse comparative sequence analysis (GRCh38/GRCm38) shows that the 5' and 3' exon blocks (*yellow arrows*) originated as a single ~89 kbp segmental duplication mapping to human chromosome 1p22.3. It was duplicated in the common ape ancestor (21 mya) from chromosome 1p22.3 to chromosome 1q21.1 in close proximity to the *NBPF* core duplicon and the promoter-containing segment of *HYDIN2* (*green arrows*). Approximately 3.2 mya, an inverted duplication (109 kbp) occurred with *NBPF* cores defining the breakpoints at chromosome 1q21.1 and 1q21.2. This was followed by the insertion of the 364 kbp *HYDIN* segmental duplication from chromosome 16q22.2. See Additional file 1: Figure S2 for phylogenetic analyses. **e** Miropeats (s = 800) schematic shows the genomic organization of the segmental duplications and surrounding gene annotation. This includes the 364 kbp *HYDIN* segment (*blue*), the 89 kbp exon-containing segment from chromosome 1p22.3 (*yellow*), and the larger, 109 kbp, inverted segmental duplication, shared with between chromosome 1q21.1 and chromosome 1q21.2 (*green* and *yellow*). *Inset* shows DHS data for fetal brain in the ~14 kbp surrounding the first exon of *HYDIN2*. The new promoter (*bent arrow*) corresponds to a peak of chromatin accessibility (see also Additional file 1: Figure S6, Table S8)

The ancestral *HYDIN* gene is notable for its large size—its canonical gene structure occupies 423 kbp of genomic sequence and by homology to mouse is predicted to produce a 15,179 bp transcript encoding a 5121 amino acid protein (Fig. 1a). Comparative sequencing of both human *HYDIN* paralogs as well as the putative integration or acceptor site in chimpanzee shows that the duplicated sequence is 364 kbp long and shares 99.4% nucleotide identity with its ancestral paralog. The duplication includes 79 coding exons but excludes the sole promoter, as well as the canonical polyadenylation site, though shorter isoforms of *HYDIN* with earlier polyadenylation sites are recorded. For transcription to occur, this gene segment must have acquired a new promoter and at least one novel polyadenylation site, potentially from flanking sequences.

The ancestral site on chromosome 16q22.2 is flanked by unique sequence, with no indication of a predisposition to rearrangement. In contrast, the acceptor site occurs within a large segmental duplication block on chromosome 1q21 that has been subject to extensive duplication and rearrangement over the last 25 million years of evolution [6, 12, 16, 19]. This includes the hyper-expanded core duplicon for chromosome 1 that carries members of the neuroblastoma breakpoint family (*NBPF*) and its associated DUF1220 protein domain [27–29]. Copies of *NBPF* map 36 kbp downstream of the insertion site and represent the nearest protein-coding gene (Fig. 1a, see also Additional file 1: Figure S1). We refined the breakpoint of the duplication integration by targeted sequencing of chimpanzee bacterial artificial chromosome (BAC) clones. A comparison with the high-quality chimpanzee sequence indicates that the insertion at chromosome 1q21.1 occurred at the boundary between LTR and LINE repeats, with concomitant loss of 841 bp of LINE sequence (Fig. 1b) in the human lineage.

To estimate the evolutionary age of the *HYDIN* duplication, we generated high-quality sequence data for the two human *HYDIN* paralogs and built a multiple sequence alignment (MSA) using orthologous sequences from chimpanzee (panTro4) and orangutan (ponAbe2) over the 364 kbp duplicated region. Phylogenetic analysis predicts that the duplication occurred approximately 3.17 million years ago (mya; 95% CI: 3.09-3.24 mya, bootstrap method) (Fig. 1c)—a period corresponding to the transition between australopithecines and the genus *Homo*. The derived *HYDIN2* duplication inserted into evolutionarily older segmental duplications shared among great apes (summarized in Fig. 1d). Immediately flanking the large central segment are two blocks, referred to here as the 5' exon block (41 kbp) and the 3' exon block (24 kbp) because they provide exons that form fusion transcripts by joining with exons from within the *HYDIN2* duplication. These two blocks were formerly a single segment that was bisected by the insertion of the duplication from chromosome 16.

The segment composed of the 5' exon block and 3' exon block maps to two other locations in the human genome in addition to chromosome 1q21.1 (Fig. 1d): an inversely oriented copy mapping 1.1 Mbp telomerically at chromosome 1q21.2 and another mapping at chromosome 1p22.3. Only the chromosome 1p22.3 locus shares conserved synteny with mouse, where it is found as a single copy and likely represents the ancestral locus. The other two copies on chromosome 1q21 are both associated with *NBPF* and form a larger homologous segment of ~109 kbp in length. Phylogenetic reconstruction predicts that the first duplication occurred ~21 mya (95% CI: 20.6–21.6 mya, bootstrap method; Additional file 1: Figure S2a), placing the event at the root of the ape lineage after divergence from the Old World monkeys [30]. This is consistent with our observation that this segment is found at single copy in New World and Old World monkeys and at two copies in gibbon and orangutan.

The second duplication, from chromosome 1q21.1 to chromosome 1q21.2, shows greater sequence identity (99.6%) than the *HYDIN* duplication (99.4%). Although phylogenetic timing predicts a more recent origin for this duplication (2.31 mya; 95% CI: 2.17–2.45 mya; Additional file 1: Figure S2b) than for the *HYDIN* duplication, it must necessarily have occurred prior to or together with the insertion of the *HYDIN* segment from chromosome 16 in order for it to have been disrupted by the insertion. It is likely that interlocus gene conversion between human chromosome 1q21.1 and chromosome 1q21.2 copies distorts the timing of this duplication. Longer stretches of conserved synteny between sequenced non-human primate clones and human chromosome 1q21.1 than between the clones and human chromosome 1q21.2 indicate that chromosome 1q21.1 is the more likely ancestral locus. This second duplication extends further upstream to include a segment that provides the new promoter (26 kbp) and further downstream to the segment that includes *NBPF12*. Altogether, the *HYDIN* segment is sandwiched by two layers of duplications—the first 21 million years old, and the other 3.2 million years old—providing the genomic substrates for novel *HYDIN2* flanking exons and regulatory elements (Fig. 1e).

### Copy number diversity and gene conversion in human populations

**Fig. 2:**
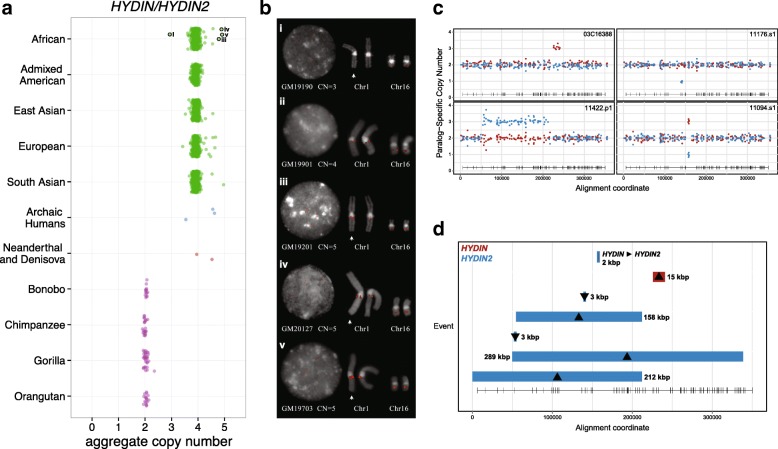
*HYDIN* copy number diversity in humans and great apes. **a** Diploid aggregate copy number for *HYDIN* loci based on genome sequencing data from 1KG (611 Africans, 285 admixed Americans, 400 East Asians, 376 Europeans, 451 South Asians), as well as archaic genomes and great apes (14 bonobos, 23 chimpanzees, 32 gorillas, 17 orangutans). Copy number was estimated using average sequence read depth across the 364 kbp segmental duplication as described previously and represents the aggregate diploid copy number for *HYDIN* and *HYDIN2*. **b** FISH analysis of metaphase and interphase chromosomal preparations from four human outliers (enumerated in panel (**a**)) and one control (aggregate cn = 4 copies) confirms rare duplications (aggregate cn = 5 copies) and losses (aggregate cn = 3 copies) of *HYDIN* restricted to *HYDIN2* on chromosome 1q21.1. **c** MIP-based genotyping of paralog-specific copy number identifies partial duplications (*top* and *bottom left panels*), deletions (*top right panel*), and putative interlocus gene conversion events (*bottom right panel*). Each point estimates paralog-specific copy number (*red*, *HYDIN*; *blue*, *HYDIN2*) based on sequencing read depth over SUNs that distinguish *HYDIN* paralogs. A total of 153 MIPs were used for genotyping and events were detected by an automated caller. Also shown is the canonical *HYDIN* gene model (*bottom* of each *plot*). **d** Summary of *HYDIN* internal structural variation and interlocus gene conversion events based on MIP genotyping of 6055 humans. Duplications (*up arrows*), deletions (*down arrows*), and the sole interlocus gene conversion event (*horizontal arrow*) are colored according to locus (*red*, *HYDIN*; *blue*, *HYDIN2*) and their spatial extent shown with respect to exonic structure (*bottom of plot*)

We assessed *HYDIN* copy number variation across diverse human populations, archaic hominins, and non-human primate genomes by applying whole-genome shotgun sequence detection and singly unique nucleotide k-mer (SUNK) analysis to obtain aggregate copy number estimates [13]. In contemporary human populations, we observe a narrow distribution of *HYDIN* copy number, centered on individuals having two diploid copies of each paralog for a total of four aggregate copies (Fig. 2a). As expected from our phylogenetic analysis, all non-human primates have just two diploid copies of *HYDIN*. Neanderthal and Denisova are believed to have diverged from modern humans approximately 700,000 years ago [31–33]. Consistent with this, we observe the duplication in their genomes as well as in three archaic human genomes (~7000–45,000 years old), though with a greater variability, possibly due to the relatively lower coverage and/or quality of these genomes [34, 35]. In total, our analysis of 2401 human genomes from the 1000 Genomes Project (1KG) and the Human Genome Diversity Project (HGDP; Additional file 1: Figure S3), indicate that the duplication is nearly fixed (99.9%) for diploid copy number in contemporary humans.

For five human samples that showed copy number variation, we performed fluorescent in situ hybridization (FISH) on chromosomal metaphase spreads for validation and cytogenetic characterization (Fig. 2b). In all instances, rare copy number variation is restricted to the duplicate copy, *HYDIN2*, on chromosome 1q21.1. We designed an orthogonal method to assay *HYDIN* paralog-specific copy number at a finer scale by designing molecular inversion probe (MIP) assays [36] to  >153 nucleotide differences that distinguish the *HYDIN* paralogs. We genotyped 6055 DNA samples from controls as well as patients with neurodevelopmental and autism spectrum disorders (Additional file 1: Table S1). Other than patients with the 1q21 microdeletion/microduplication syndrome, copy number variation of *HYDIN2* was rare. The assay confirmed rare deletions and duplications of *HYDIN2* (described above) as well as rare copy number variants in *HYDIN* previously detected by array comparative genomic hybridization (CGH) of autism patients [37]. We discovered additional rare internal duplications and deletions, ranging in size from 3 kbp to 289 kbp, affecting both *HYDIN* paralogs (Fig. 2c and d; see also Additional file 1: Figure S4 and Additional file 1: Table S2). Remarkably, two control individuals showed clear signatures of interlocus gene conversion over a common ~2 kbp region. This event is copy number neutral but clearly shows that *HYDIN* has served as the donor for the conversion of sequence to *HYDIN2*. Such events are rare (2/2981 or 0.00067) but indicate non-reciprocal sequence exchange has occurred between *HYDIN* paralogs on chromosomes 1 and 16.

In summary, we observe no heterozygous deletions in *HYDIN* in the 1KG or the HGDP genome samples. Pathogenic mutations in the ancestral copy of *HYDIN* have been observed only in the homozygous state in consanguineous families [26]. The *HYDIN* duplications (2) and deletions (1) that have been observed in autism cases are large and include other genes [37]. *HYDIN2* copy number variation occurs rarely and is largely restricted to individuals carrying chromosome 1q21.1 rearrangements. We have observed no individuals with a loss of both copies of *HYDIN2*.

#### *HYDIN2* fusion transcripts

**Fig. 3:**
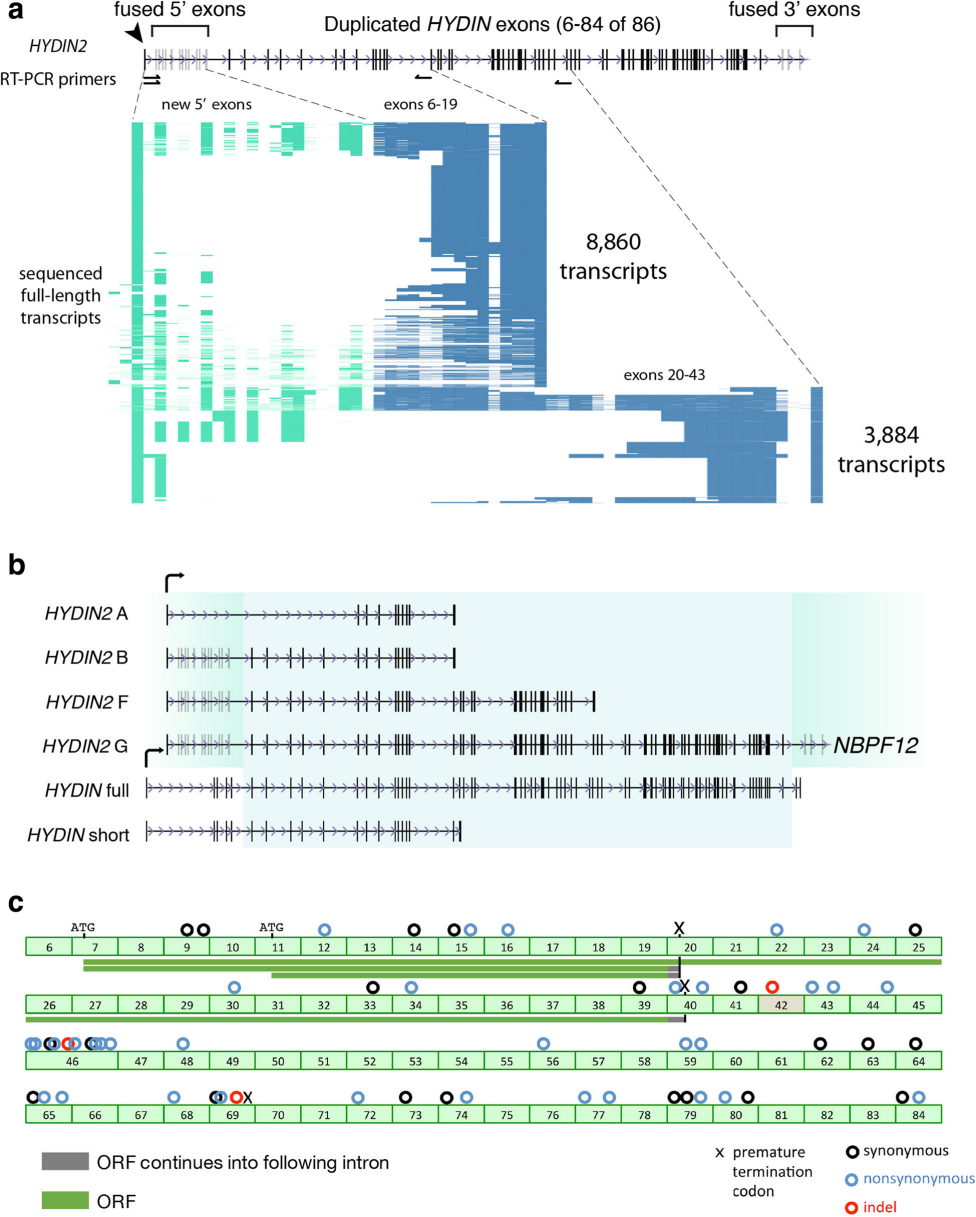
*HYDIN2* transcript diversity and ORF potential. **a** Long-range RT-PCR amplicons spanning the first exon identified by 5' RACE to putative terminal exons 19 and 43 (primer pairs shown as *half arrows*) were targeted for long-read single-molecule sequencing. A total of 12,744 amplicons were characterized and isoform content visualized for each product (*row*), with exons (*columns*) colored based on whether they are part of the canonical *HYDIN* gene structure (*blue*) or exapted from flanking sequence (*green*). **b** *HYDIN* and *HYDIN2* transcript isoforms based on long-read sequencing of RT-PCR products. Exons corresponding to the duplicated segment (*blue shading*) and flanking sequences for *HYDIN* (*white*) and *HYDIN2* (*green*). Three *HYDIN2* isoforms were identified (isoforms A, B, and F) and an isoform that spans the segmental duplication on both sides (isoform G) was constructed from multiple overlapping reads. The full-length, canonical *HYDIN* (ENST00000393567.6) and its shorter isoform (ENST00000321489.9) are shown. Exons in *gray* are subject to alternative splicing. **c** Predicted ORFs for *HYDIN2* (*green bars*) are shown with respect to *HYDIN* gene structure. Coding differences are indicated above exons, numbered with respect to the canonical isoform of the ancestral gene. *Circles* indicate synonymous (*black*), non-synonymous (*blue*), and indel (*red*) differences. Note: a 2095 bp *HYDIN2* deletion eliminates part of the intron 41 and exon 42, including the splice acceptor for exon 42. Exon 42 is skipped and exon 41 is rarely observed in *HYDIN2* transcripts. Productive *HYDIN2* transcripts are unlikely to continue past exon 42. The three longest ORFs are predicted to be 1852 aa (Isoform F, exons 7-39), 668 aa (Isoform B; exons 7-19) and 467 aa (Isoform A, exons 11-19); only Isoform A lacks multiple exons 5′ to the ORF

We investigated the gene structure of *HYDIN2* by first considering an alignment of the theoretical open reading frame (ORF) based on the ancestral *HYDIN* gene model (Fig. 3). If all duplicated codons were maintained as in *HYDIN*, the theoretical alignment would yield 21 synonymous and 32 non-synonymous differences between the shared sequence, as well as three deletions (Fig. 3c). The latter includes: a 2095 bp deletion (with intronic sequence) on *HYDIN2* that eliminates the splice acceptor for exon 42, a 15 bp in-frame deletion in exon 46, and a 1 bp deletion in exon 69. Due to the deletion of exon 42 and the frameshift in exon 69, a premature stop codon is predicted. As a result of the incomplete gene structure with respect to *HYDIN*, *HYDIN2* is annotated as a pseudogene by both RefSeq and GENCODE.

We identified mapped expressed sequence tags (ESTs), both within and spanning the *HYDIN* duplication breakpoints, as evidence that transcription might still be occurring, potentially by way of gene fusion to neighboring sequence. To identify the new transcriptional start site, we performed 5' rapid amplification of complementary DNA (cDNA) ends (5' RACE), using fetal brain RNA as starting material. We identified a site 55 kbp upstream, which we define as the *HYDIN2* promoter. The high density of spliced ESTs at this location supports this as a site of transcription initiation; we also observe a robust fetal brain DNase1 hypersensitivity peak (Fig. 1e, Additional file 1: Figure S6) consistent with its function as an alternate promoter. Although duplication of *HYDIN* excluded the canonical polyadenylation site of the longest *HYDIN* isoform, shorter isoforms of *HYDIN* that terminate at exons 15, 19, and 20 are also annotated. We identified spliced ESTs supporting exon 19 as an alternative site of polyadenylation in *HYDIN*. We further investigated alternative polyadenylation through 3' RACE and identified a potential polyadenylation site at exon 43 of *HYDIN2*.

We amplified by RT-PCR the putative full-length transcripts that spanned from the new *HYDIN2* promoter to both the polyadenylation site at exon 19 and the polyadenylation site at exon 43, using fetal brain RNA as a starting material (Fig. 3a). We also designed a series of smaller RT-PCR products extending into the fused 3′ exons. Products were sequenced with single-molecule, real-time (SMRT) sequencing technology, which allowed us to resolve patterns of splicing, although we note that abundance of different isoforms cannot be taken as evidence of messenger RNA (mRNA) expression levels due to the preferential loading bias for smaller isoforms inherent to the sequencing technology. We identified fusion transcripts with both upstream and downstream segments beyond the *HYDIN* duplication breakpoints. Upstream, these transcripts begin at the promoter identified by 5' RACE and continue into the *HYDIN* duplication. We observe 13 5'-fused exons, with considerable diversity in alternative splicing. The transcripts that begin with these exons continue into the *HYDIN* duplication and then generally follow the canonical pattern of *HYDIN* splicing. Downstream, we also identify fusion transcripts between the *HYDIN* duplicate exons and the 3′ exon block. These fusion transcripts continue into the neighboring gene, *NBPF12*, a gene that itself undergoes highly variable splicing, with annotated transcripts in the range of 1831–7061 bp in length.

Transcripts that begin at the new promoter and continue into the duplicated *HYDIN* exons are predicted to initiate translation consistent with the *HYDIN* ORF at the ATG located in exon 7 or an alternate ATG mapping to exon 11 (Fig. 3c). Based on sequencing of transcripts, we predict ORFs of 467 and 668 amino acids (transcript termination at exon 19) and an ORF of 1852 (transcript termination at exon 43). These transcripts are designated *HYDIN2* isoforms A, B, and F, respectively (Fig. 3b). If translated, these products would represent truncated HYDIN proteins (*green bars* in Fig. 3c). The 5' and 3' fusion exons (located in *NBPF12*) do not extend ORFs beyond the *HYDIN* duplication. We do not find evidence of internal alternative sites of transcription initiation, whether by ESTs, cap analysis gene expression (CAGE), or 5' RACE, although RACE has not been performed exhaustively throughout the 364 kbp duplicated segment.

### Expression analysis

**Fig. 4:**
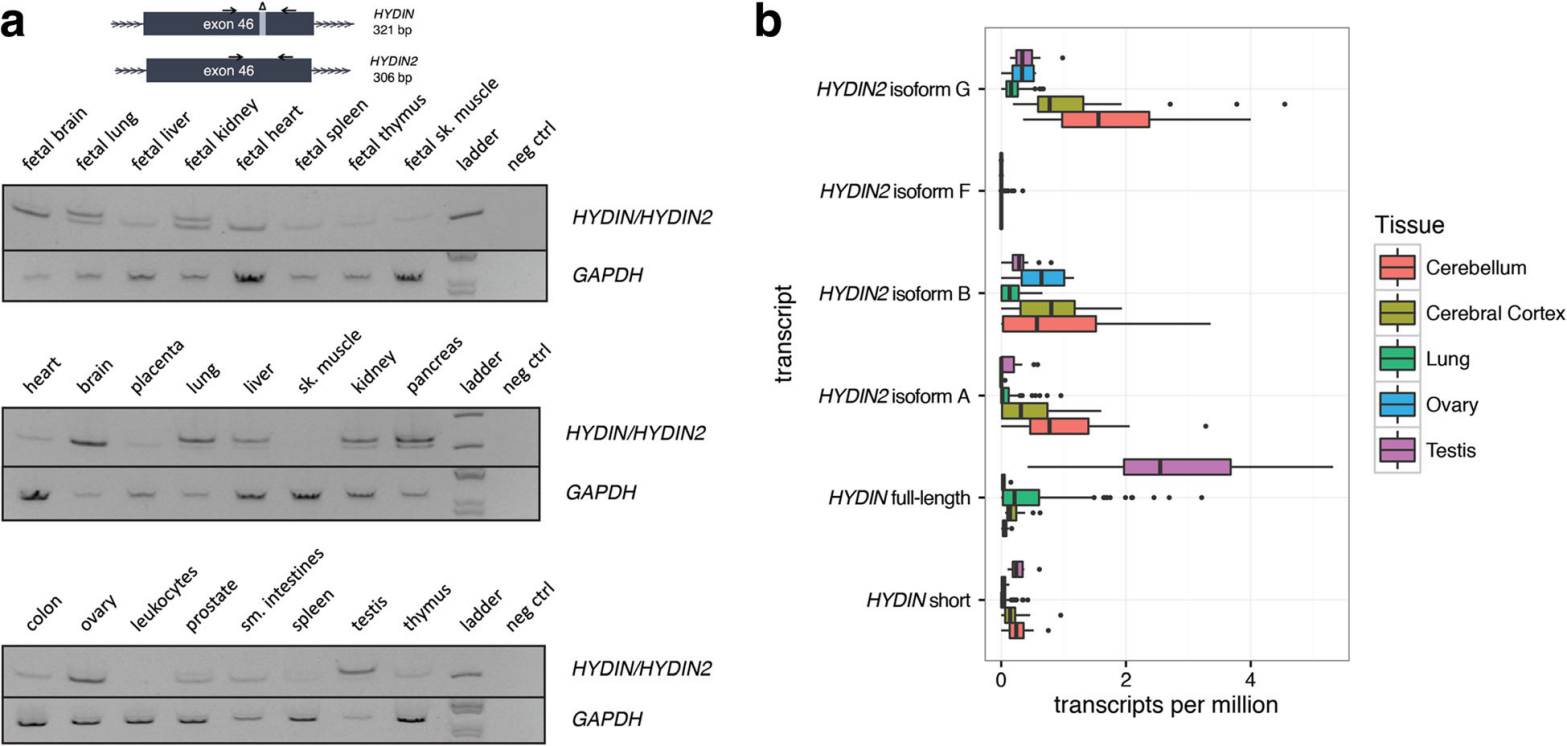
Tissue-specific expression of *HYDIN/HYDIN2* isoforms. **a** RT-PCR analysis over a 15 bp deletion in exon 46 of *HYDIN2* compares the relative abundance of *HYDIN* (*top band*, 321 bp) and *HYDIN2* (*bottom band*, 306 bp) mRNA in different adult and fetal tissues. Images have been inverted and brightness adjusted for clarity. Adult brain and ovary show the highest levels of *HYDIN2* while *HYDIN* is expressed predominantly in lung, pancreas, and testis. In fetal tissues, *HYDIN2* is expressed more ubiquitously. **b** RNA-seq reads from various tissues (The GTEx Consortium, 2013) containing SUNKs (k = 30) were used to estimate *HYDIN* and *HYDIN2* expression. Full-length *HYDIN* is expressed most highly in the lung and testis, while all isoforms of *HYDIN2* are more highly expressed in brain tissues. Isoform F is not expressed at a level that is likely to be significant. *Boxplots* indicate median and interquartile range (IQR) with outliers shown beyond 1.5 × IQR

Our results suggest that *HYDIN2* acquired a new promoter and we hypothesize that the novel promoter is responsible for the expression differences between the paralogs. We took advantage of a 15 bp in-frame exonic deletion that distinguishes *HYDIN2* from *HYDIN* to investigate the transcript abundance in different tissues (Fig. 4a). By designing RT-PCR primers that flanked this deletion, we inferred relative expression of the *HYDIN* paralogs from the differences in signal intensity. Next, we leveraged RNA sequencing (RNA-seq) reads that could be mapped to SUNK differences between *HYDIN* and *HYDIN2* to quantify relative expression levels across a panel of tissues using data from the GTEx project [38] (Fig. 4b). The latter analysis allowed us to compare expression among the different isoforms characterized by cDNA sequencing as well as quantify differences between paralogs.

We observe that *HYDIN* is most highly expressed in ciliated tissues, such as the lung and testis, consistent with its known function as a ciliary structural protein [39]. By comparison, *HYDIN2* expression is higher in the brain, while also prominent in the ovary. Interestingly, *HYDIN2* appears more broadly expressed in fetal tissues with the strongest signal observed in fetal brain. It should be noted that the RT-PCR assay measures expression of exon 46 alone, which is not included in some shorter RefSeq isoforms of *HYDIN*. Comparison of this assay with SUNK-based mapping of the GTEx data is in close agreement. This is also consistent with the observation that cDNA clones from *HYDIN2* were predominantly derived from neuronal sources [24]. With the exception of *HYDIN2* isoform F, all three isoforms show moderate levels of expression in cerebellum and cerebral cortex. The longest isoform, *HYDIN2* isoform G, with both 5' and 3' fused exons shows a slightly higher level of expression, although we note only that only *HYDIN2* isoform A produces a transcript without a large number of 5' untranslated exons due to exon skipping from the first exon to exon 11 of the canonical gene model where an alternate ATG start codon exists.

The predicted *HYDIN2* promoter overlaps a DNase I hypersensitivity (DHS) peak (Fig. 1e, Additional file 1: Figure S6) in fetal brain. A number of fetal brain cDNA sequences have been mapped to the duplicated locus of the promoter-containing block on chromosome 1q21.2 [40, 41]. Notably, at the ancestral chromosome 1p22.3 locus (Fig. 1d), where the 5' and 3' exon blocks sit in the absence of the promoter, there are neither annotated transcripts nor spliced ESTs. Within the DHS peak a smaller, higher copy repeat is found, approximately 1113 bp long. A simple BLAT search reveals that this segment has propagated throughout chromosome 1, with ten locations that contain the full-length repeat at a level of identity of 90% or above (Additional file 1: Table S3). Many are clustered at chromosome 1q21 and are found in association with, though not transcriptionally joined to, the *NBPF* core duplicon. Eight out of ten produce spliced ESTs, many from neuronal tissues, further supporting this segment as contributing to the expression pattern of *HYDIN2*.

### Coding variation and selection in *HYDIN* and *HYDIN2*

We investigated whether there was evidence of selection acting on *HYDIN2* by comparing the ratio of non-synonymous to synonymous changes between paralogs, using non-human primate *HYDIN* as an outgroup (Additional file 1: Table S4). We observe moderately strong purifying selection acting on ancestral *HYDIN* throughout the primate lineage, with an average dN/dS value of 0.29. *HYDIN2*, in contrast, shows an elevated pairwise dN/dS value in comparison with non-human primates (e.g., 0.39 for human *HYDIN2* and chimpanzee *HYDIN* versus 0.29 for human *HYDIN* and chimpanzee *HYDIN*). Branch-based estimates of dN/dS that can detect adaptive evolution after gene duplication [42] show a similar trend with a consistently elevated dN/dS value for human *HYDIN2* when compared to human *HYDIN*. This result holds if we restrict our analysis to only those exons predicted to be part of the *HYDIN2* ORF (*HYDIN2* isoform A). Although none of these differences achieve statistical significance due to the limited number of mutational differences occurring within the human lineage, it is interesting that all three mutational changes that occurred within the *HYDIN2* ORF result in amino acid changes while only synonymous changes (n = 2) occurred in the corresponding portion of ancestral *HYDIN*.

As an alternative approach, we sought to characterize and compare deleterious coding variation between *HYDIN* and *HYDIN2* among 3484 probands and 2629 healthy controls from families with autism [43, 44]. We targeted all coding exons with at least five nucleotides of flanking sequence based on the canonical *HYDIN* gene structure using MIPs. We identified all likely gene-disruptive (LGD) variants (frameshift, stop-gain, stop-loss, or splice-site mutations) and successfully assigned 39% of such deleterious variants to either *HYDIN* or *HYDIN2*, made possible by singly unique nucleotides (SUNs) contained within the target sequence of the MIP (Table 1). No common (>1% allele frequency) LGD variants were observed for either paralog. Considering the canonical ancestral gene structure, we initially observed a lower number of LGD mutations for *HYDIN* (n = 2) when compared to *HYDIN2* (n = 10) in controls. Interestingly, if we restrict our analysis to the most likely ORF model (*HYDIN2* isoform A), only two *HYDIN2* LGD mutations remain and at an allele frequency comparable to what has been observed for *HYDIN.* Assuming that all unassigned LGD mutations originate from *HYDIN2*, all higher frequency LGD mutations (>0.15% frequency) correspond to ancestral exonic sequence mapping outside of the *HYDIN2* ORF gene model (Additional file 1: Table S5).

**Table 1:** Likely gene-disruptive events detected in *HYDIN*/*HYDIN2* by MIP-based sequencing of exons in cases and controls

Paralog^a^	Variant	Exon	Intron	Protein position	Amino acid	Cases (n = 3483)			Controls (n = 2629)		
						n	Freq.	Number genotyped^b^	n	Freq.	Number genotyped^b^
Cases only											
*HYDIN2*	splice_donor	-	66/85	-	-	1	0.03%	3431	0	0.00%	2604
*HYDIN2*	splice_acceptor	-	53/85	-	-	1	0.03%	3432	0	0.00%	2599
*HYDIN*	stop_gained	48/86	-	2690	Q/*	1	0.03%	3433	0	0.00%	2603
*HYDIN2*	stop_gained	46/86	-	2540	R/*	1	0.03%	3425	0	0.00%	2598
Controls only											
*HYDIN2*	stop_gained	80/86	-	4563	W/*	0	0.00%	3429	1	0.04%	2599
*HYDIN2*	Frameshift	48/86	-	2680	G/X	0	0.00%	3427	1	0.04%	2598
*HYDIN2*	splice_donor	-	42/85	-	-	0	0.00%	3428	1	0.04%	2599
*HYDIN*	splice_donor	-	29/85	-	-	0	0.00%	3434	1	0.04%	2603
*HYDIN*	stop_gained	11/86	-	1330	R/*	0	0.00%	3429	1	0.04%	2599
Found in both cases and controls											
*HYDIN2*	splice_acceptor	-	67/85	-	-	11	0.32%	3430	6	0.23%	2601
*HYDIN2*	splice_acceptor	-	54/85	-	-	1	0.03%	3426	1	0.04%	2595
*HYDIN2*	frameshift	46/86	-	2485	A/X	1	0.03%	3428	1	0.04%	2600
*HYDIN2*	frameshift	41/86	-	2115-2116	VI/VSX	11	0.32%	3427	10	0.38%	2598
*HYDIN2*	splice_donor	-	28/85	-	-	1	0.03%	3430	1	0.04%	2600
*HYDIN2*	frameshift	19/86	-	2531-2532	A/X	1	0.03%	3434	1	0.04%	2607
*HYDIN2*	splice_acceptor	-	14/85	-	-	4	0.12%	3429	2	0.08%	2605

We genotyped 3483 probands and 2629 healthy controls from families with autism using a MIP-based genotyping assay that targeted coding exons and at least five flanking intronic nucleotides. LGD variants (frameshift, stop-gain, stop-loss, and splice-site) were called using FreeBayes. Only variants that could be definitively assigned to *HYDIN* or *HYDIN2* based on the presence of an identifying SUN are shown. Variants include those seen only in cases, seen only in controls, and those seen in both cases and controls. Most of the variants seen in *HYDIN2* occur outside of the putative coding sequence

^a^Paralog determined by presence of SUN(s) on variant-containing MIP reads; variants identified by MIP reads that did not intersect a SUN could not be assigned. Variants in *HYDIN2* are annotated with the exon numbering scheme from *HYDIN*

^b^Number of samples successfully genotyped for this variant (FreeBayes)

### *HYDIN2* and the chromosome 1q21 microdeletion/microduplication syndrome

**Fig. 5:**
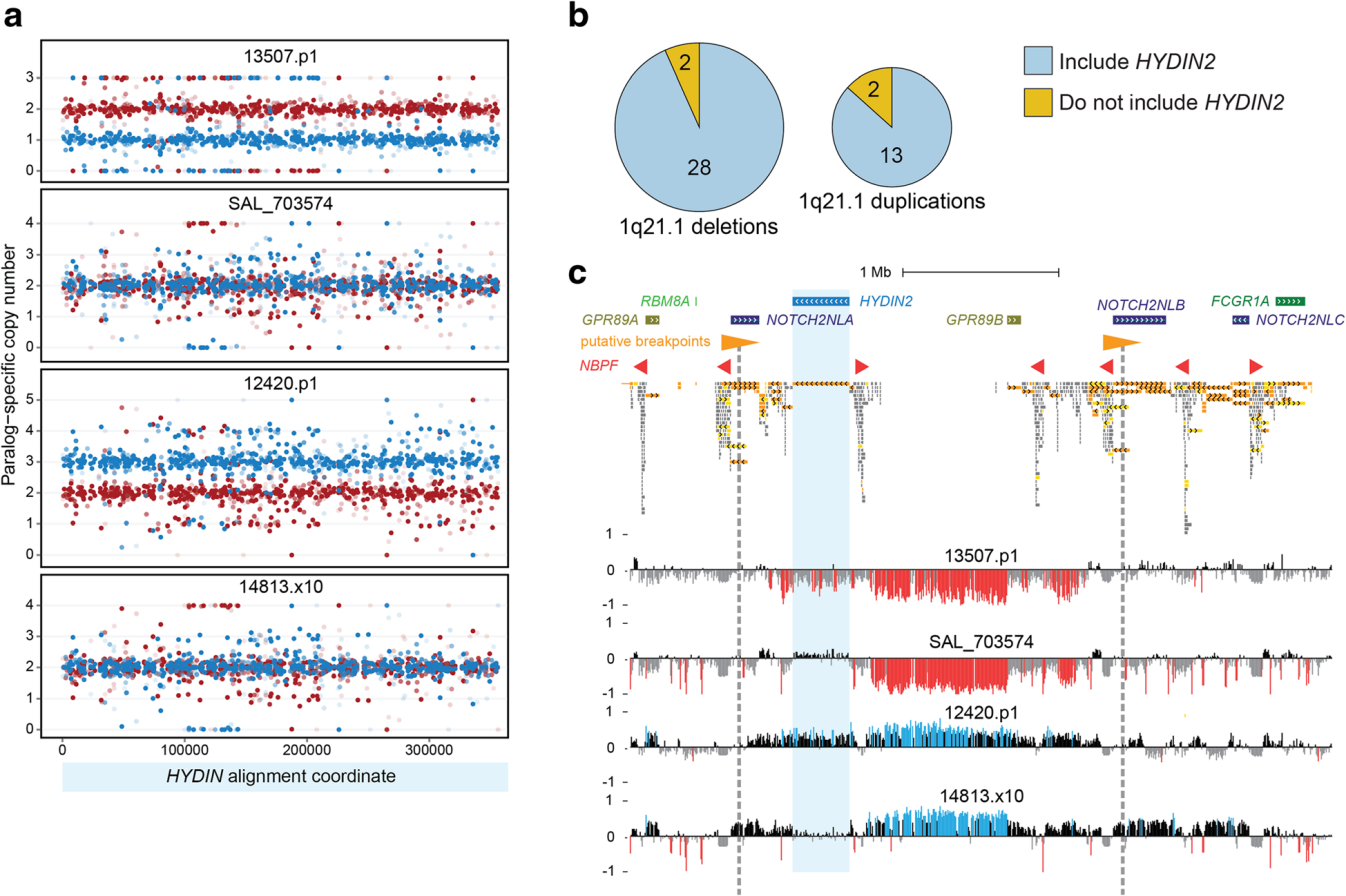
*HYDIN2* and chromosome 1q21 rearrangement breakpoint variability. We genotyped 73 patients carrying either the chromosome 1q21 microdeletion (n = 48) or microduplication (n = 25) for *HYDIN2* copy number using MIPs. **a** Patients were genotyped using 717 MIPs targeting variants that distinguish *HYDIN* paralogs. Points show *HYDIN* paralog-specific copy number estimates (*red*, *HYDIN*; *blue*, *HYDIN2*) for two microdeletion (13507.p1 and SAL_703574) and two microduplication (12420.p1 and 14813.x10) patients. Patients 13507.p1 and 12420.p1 show deletion and duplication of *HYDIN2*, respectively, while SAL_703574 and 14813.x10 do not. **b** Summary of results across 45 independent microdeletion and microduplication events from 73 individuals based on MIP sequencing and analysis. Approximately 91% of 1q21 rearrangements examined include *HYDIN2*. **c** Array CGH results confirm 1q21 rearrangements in the samples in panel a and copy number changes of *HYDIN2* (*blue shading*) only in patients 13507.p1 and 12420.p1. Note: log2 hybridization signal intensity (*y-axis*) values are depressed when compared to unique sequence due to duplicated nature of sequence (*red* = deletion signal; *blue* = duplication signal). Results are shown for a 4.5 Mbp region at chromosome 1q21 (GRCh38 chr1: 145,500,001–150,000,000) with genes and segmental duplications annotated (*orange* = 99% sequence identity or above; *yellow* = 98–99%; *gray* = 90–98%). *Orange triangles* indicate high-identity, directly oriented *NOTCH2NL-NBPF* duplications, with putative breakpoints of the canonical 1q21 rearrangement shown as *vertical gray dashed lines*. Shown below are the locations of *NBPF* core duplicons

Recurrent microdeletions and microduplications at chromosome 1q21 have been associated with a variety of neurodevelopmental phenotypes, including microcephaly and macrocephaly. Loss or gain of *HYDIN2* has been hypothesized to underlie these head circumference phenotypes in light of its expression in brain, its inclusion in the typical rearrangement interval, and the association of homozygous losses of *HYDIN* in mouse with hydrocephalus [22, 24, 25]. To explore this hypothesis, we leveraged our MIP assay (Fig. 5a) to genotype *HYDIN* paralog-specific copy number in 73 individuals carrying a chromosome 1q21 rearrangement, corresponding to 45 independent rearrangement events (15 duplications and 30 deletions). MIP data revealed that *HYDIN2* is usually, but not always, affected by chromosome 1q21 deletions and duplications (Fig. 5b, Additional file 1: Figure S5). Overall, we observe that 87% of duplications (13 of 15) and 93% of deletions (28 of 30) examined include *HYDIN2*. Targeted array CGH on a subset of patients validated our whole-genome-shotgun-based and MIP-based results in every instance, confirming inclusion or exclusion of *HYDIN2* among both 1q21 microduplications and microdeletions (Fig. 5c, Additional file 1: Figure S5).

We also performed comprehensive segmental duplication analysis on the GRCh38 reference haplotype to identify directly oriented duplication pairs with high sequence identity that may confer susceptibility to non-allelic homologous recombination. For typical 1q21 rearrangements, those that include *HYDIN2*, the most likely candidates are large (247 kbp), highly identical (99.7%) segments that include truncated *NOTCH2* duplications (*NOTCH2NL*) adjacent to members of the *NBPF* gene family (Fig. 5c, Additional file 1: Figure S5b). For atypical rearrangements, our data are consistent with multiple possible breakpoint locations. Alternatively, these events may have occurred on a still undescribed haplotype structure or originated through a nonrecurrent mechanism.

We examined the phenotype of atypical carriers without copy number variation in *HYDIN2*. All three atypical 1q21 microduplications excluding *HYDIN2* present with macrocephaly and similarly all three patients with atypical 1q21 microdeletions excluding *HYDIN2* exhibit microcephaly (Additional file 1: Table S6). These observations suggest that loss or gain of a genomic *HYDIN2* copy is not necessary for chromosome 1q21 rearrangement patients to manifest head circumference abnormalities. We cannot rule out the possibilities that these individuals harbor disruptive point mutations in *HYDIN2* or that atypical chromosome 1q21 rearrangements dysregulate *HYDIN2* expression. Further studies will be necessary to elucidate the potential role of *HYDIN2* in brain size and in other aspects of chromosome 1q21 rearrangement phenotypes.

## Discussion

*HYDIN2* is a human-specific gene that emerged 3.2 mya, created by incomplete duplication of the ancestral gene *HYDIN*. Young duplicate genes can rapidly evolve essential functions [45]; however, unless increased gene dosage is itself beneficial, long-term maintenance of a duplicate gene typically requires mutational change leading to functional innovation or subfunctionalization [46]. Incomplete gene duplication provides one mechanism for rapid functional change because the duplicate differs in structure from its progenitor, with potentially profound functional consequences beyond a simple dosage increase. Such a mechanism has been postulated in the case of the human-specific incomplete duplications of *SRGAP2*, where the truncated granddaughter paralog, *SRGAP2C*, has been shown to antagonize the ancestral copy *SRGAP2A* [14, 16].

Interspersed duplications such as *HYDIN* have an additional mechanism promoting functional divergence, namely, duplicate copies in new genomic locations are subjected to asymmetric rates of mutation [47]. Because the *HYDIN* duplication excluded the promoter, naively one would expect the duplicate copy to have been silenced. In contrast, our analysis shows that *HYDIN2* is actively transcribed, and more highly expressed than the ancestral paralog in many tissues. The acquisition of a novel promoter effectively created a fusion gene. Thus, the partial duplication was “rescued” by its juxtaposition with active regulatory sequence at chromosome 1q21.1.

The original function of this novel neuronal promoter is not clear, but 2 Mbp away at chromosome 1q21.2 sits an earlier duplication of the *HYDIN2* flanking sequences without the *HYDIN2* insertion. Here, we observe robust transcription, as evidenced by a number of GENCODE transcripts—all classified as long non-coding RNA and most of which derive from a fetal brain cDNA library [41]. At *HYDIN2*, this promoter has driven an altered expression pattern, with widespread expression in fetal tissues, decreased expression in testis and lung, and increased expression in brain tissues (cerebellum and cerebral cortex) and ovary being the most prominent changes.

Long-read sequencing of cDNA from both the fetal and adult brain reveals an extraordinary diversity of *HYDIN2* isoforms, including the presence of additional 5' and 3' exons within transcripts spanning the duplication junctions. Although we can confirm expression of at least three distinct *HYDIN2* isoforms, we favor isoform A as the most likely protein-encoding transcript for several reasons: there is no evidence for a premature termination codon; deleterious coding mutations in the human population are rare; and the other isoforms carry an unusually large number of untranslated exons. The presence of abnormally long untranslated regions (UTRs) or exon junctions downstream of a premature termination codon usually indicates strong signatures for nonsense mediated decay of mRNA [48]. Similarly, a large number of 5' non-coding exons is thought to impede translational efficiency [49, 50].

In the case of *HYDIN2* isoform A, intervening untranslated exons are skipped, resulting in a putative 467 amino acid protein with relatively short 5' and 3' UTRs. Although we know little regarding the function of *HYDIN2*, it is noteworthy that *HYDIN* has a structural role in motile cilia [39, 51, 52]. Its expression in lung and testis is consistent with the observation that recessive mutations in *HYDIN* cause primary ciliary dyskinesia, with the primary phenotypes being chronic respiratory infections and male infertility in humans [26]. It is interesting that in addition to these deficiencies, mouse mutants in *hy3* (*Hydin* ^–/–^) show a more severe phenotype, developing lethal hydrocephalus due to impaired ciliary motility and fluid flow in the developing brain. One possible explanation for this phenotypic discrepancy between mutant mice and humans lacking functional *HYDIN* may be that human *HYDIN* paralogs have undergone subfunctionalization. In particular, the neuronally expressed *HYDIN2* may have assumed some of the ancestral gene’s function during human brain development.

Our copy number analyses reveal that the *HYDIN2* duplication has largely fixed in the human population. In fact, *HYDIN2* shows the lowest degree of copy number variation in the normal population when compared to other human-specific duplications [8, 13]. While it was speculated that *HYDIN2* played an important role in the reciprocal macrocephaly/microcephaly phenotype associated with 1q21.1 duplications/deletions [22], we have identified rearrangement patients with head size abnormalities lacking altered genomic dosage of *HYDIN2*. It is plausible that altered expression or point mutations effectively disrupt *HYDIN2* in these individuals or, alternatively, that *HYDIN2* dysfunction does not contribute to their head circumference phenotypes. Distinguishing these possibilities and determining whether *HYDIN2* plays an important role in neurodevelopment more broadly will require further functional studies complemented by large-scale genotyping in various neurodevelopmental disease cohorts and relevant, well-phenotyped controls.

## Conclusions

In this study, we characterize the evolutionary history, transcriptional landscape, and potential clinical impact of the human-specific duplicate gene *HYDIN2*. We show that *HYDIN2* was generated by the juxtaposition of multiple segmental duplications culminating with the partial duplication of *HYDIN* ~3.2 mya. We identify a new promoter that “rescued” the truncated gene duplicate and drives a neuronal pattern of expression. We show that long-read sequencing can be used to understand a previously intractable large and complexly spliced gene and identify transcribed unannotated ORFs. We show that the reciprocal macro/microcephaly phenotypes associated with chromosome 1q21 rearrangements can occur without *HYDIN2* copy number changes. Ultimately, we provide a clear example of how juxtaposition of transcriptionally active segmental duplications can lead to the birth of a new gene.

## Methods

### FISH

Metaphase and interphase spreads were prepared from lymphoblastoid human cell lines (GM19190, GM19901, GM19201, GM20127, GM19703; Coriell Cell Repository). FISH experiments were performed using fosmid clone WIBR2-3823 N03, directly labeled by nick-translation with Cy3-dUTP (PerkinElmer) as previously described [53] with minor modifications. Briefly, 300 ng of labeled probe was used for the FISH experiments; hybridization was performed at 37 °C in 2 × SSC, 50% (v/v) formamide, 10% (w/v) dextran sulfate, and 3 mg sonicated salmon sperm DNA, in a volume of 10 μL. Posthybridization washing was at 60 °C in 0.1 × SSC (three times, high stringency). Nuclei were simultaneously DAPI stained. Digital images were obtained using a Leica DMRXA2 epifluorescence microscope equipped with a cooled CCD camera (Princeton Instruments). DAPI and Cy3 fluorescence signals, detected with specific filters, were recorded separately as grayscale images. Pseudocoloring and merging of images were performed using Adobe Photoshop software.

### Sequencing and assembly of large-insert clones (BACs)

We searched for discordant BAC-end mappings that spanned the *HYDIN2* insertion site at chromosome 1q21.1 in libraries CH251 (chimpanzee) and CH276 (orangutan). One clone was identified for chimpanzee (CH251-231E10) and one in orangutan (CH276-57C3). DNA was isolated from these clones and SMRTbell libraries were prepped and sequenced on the Pacific Biosciences (PacBio) RSII. Inserts were assembled using HGAP and Quiver-polished as previously described [54].

### Phylogenetic analysis

Orthologous *HYDIN* sequences in chimpanzee and orangutan were identified using BLAT [55] in the UCSC Genome Browser [56]. The human chromosome 16 *HYDIN* shared sequence was used as a query against panTro3 and ponAbe2, respectively. An MSA of these non-human primates as well as the duplicated sequences from chromosomes 1 and 16 (both from CH17) was created using ClustalW [57]. An unrooted phylogenetic tree was constructed in MEGA6 [58] using the neighbor-joining method [59] with complete-deletion option, yielding a total of 315,349 positions. Genetic distances were computed under the Kimura two-parameter model [60] with standard error estimates [61] (n = 500 bootstrap replicates). A Tajima’s relative rate test using chimpanzee as the outgroup failed to reject the hypothesis that both human *HYDIN* paralogs are evolving at the same rate (*p* = 0.21). Thus, the timing of the duplication event is estimated by taking the average evolutionary distance between the two *HYDIN* paralogs as a ratio of the total distance from chimpanzee *HYDIN*. This yields a timing estimate of 3.17 mya assuming the divergence took place 6 mya. The 95% confidence interval was estimated by the bootstrapping method.

### Copy number genotyping

Aggregate and paralog-specific copy number estimates of *HYDIN/HYDIN2* were determined using previously described methods [13]. Raw sequences from 236 human individuals from HGDP [11], 2143 human individuals through Phase 3 of 1KG [10], 86 non-human primate individuals from the Great Ape Genome Project (including bonobos (n = 14), chimpanzees (n = 23), gorillas (n = 32), and orangutans (n = 17)) [62], a Denisovan individual [32], a Neanderthal individual [33], and three archaic hominids [34, 35] were mapped to the human reference genome using mrsFAST [63]. For aggregate copy number estimates (Fig. 2a) GRCh37 was used. For paralog-specific copy number estimates (Additional file 1: Figure S3) GRCh38 was used, as recent correction to the *HYDIN2* locus in the human genome made possible paralog-specific copy number estimates.

### Expression quantification

Kallisto (v. 0.42.4) [64] was used to estimate the expression levels of nine transcripts detected and putative *HYDIN2* isoforms (see Additional file 2). We added the *HYDIN2* sequences to the GENCODE reference transcriptome (release 25) [40] and generated a new index using kallisto. Transcripts per million values were then calculated using kallisto with default parameters for all of the GTEx RNA-seq samples (dbGaP version phs000424.v3.p1) from the following tissues: cerebellum (38 samples), cerebral cortex (31 samples), lung (133 samples), ovary (six samples), and testis (15 samples).

To experimentally determine relative expression of *HYDIN* and *HYDIN2* in various tissues, we took advantage of a 15 bp deletion in exon 46 of *HYDIN*. Identical flanking sites were chosen for priming, so that relative expression of transcripts containing exon 46 could be measured in a single reaction. Expected band sizes were 321 bp for *HYDIN* and 306 bp for *HYDIN2*. A total of 5 μL of cDNA from various adult and fetal tissues normalized for expression level was used as template (Clonetech Human MTC Panel I, Human MTC Panel II, Human Fetal MTC Panel I (obtained from spontaneously aborted fetuses, aged 16–40 weeks)) and PCR was performed as per manufacturer’s instructions, with GAPDH as a positive control. Reactions were monitored by the level of SYBR Green I (Invitrogen) fluorescence using Bio-Rad MiniOpticon Real-Time PCR System. Reactions proceeded for 31 cycles of amplification (19 for GAPDH reactions) and PCR products were visualized by 20 min of electrophoresis using E-Gel EX Agarose Gels (4%; Invitrogen).

### RACE experiments

5' and 3' RACE were performed using the FirstChoice RLM-RACE Kit (Ambion) as per manufacturer’s instructions using the nested protocol on poly-A^+^ RNA derived from fetal brain (Clontech). Spliced ESTs in the *HYDIN2* locus were taken as evidence of active transcription, and pileups of ESTs with shared edges were taken as evidence of potential sites of transcription initiation and termination and were chosen as targets for RACE. The ancestral paralog was also targeted as a positive control. PCR products including secondary bands were gel extracted and purified using the QIAquick Gel Extraction Kit (Qiagen) and capillary sequenced. In cases where RACE products did not include unique sequence, PCR products were purified using the QIAquick PCR Purification Kit (Qiagen) and cloned using the TOPO XL PCR Cloning Kit with OneShot TOP10 Chemically Competent *E. coli* (Invitrogen). Cells were streaked onto agar plates containing 50 μg/mL kanamycin and incubated overnight at 37 °C. Individual colonies were picked and subjected to colony PCR, where the insert was amplified using standard M13 forward and reverse primers. The PCR products were purified and sequenced as before. Unique nucleotide differences were used to infer the paralog of origin. Primers used can be found in Additional file 1: Table S7.

### PacBio cDNA sequencing

cDNA was synthesized from poly-A^+^ RNA using oligo(dT) priming either SuperScript II or SuperScript III reverse transcriptase (Invitrogen) with the following modification: an extra 50 min was added to the reverse transcription incubation time following the addition of another 1 μL of enzyme. A total of 1 μL of from the cDNA synthesis reaction was used as template for nested PCR with Kapa HiFi PCR Kit. PCR amplicons were purified using magnetic beads (Agencourt AMPure XP, 1X concentration). Library preparation for PacBio sequencing of PCR amplicons was performed using standard and approved reagents and protocols. Two SMRT cells were sequenced: the first, which included amplicons spanning from new *HYDIN2* promoter to exon 19 was run using P5C3 chemistry, the second, which included amplicons spanning from the new *HYDIN2* promoter to exon 43, as well as intermediate fragments from exon 19 through beyond the 3′ duplication breakpoint, was run using P6C4 chemistry. Primers used can be found in Additional file 1: Table S7.

### DNase I hypersensitivity (DHS) at *HYDIN2* promoter

Chromatin accessibility, as measured by DHS, was assessed for evidence of regulatory activity of the new *HYDIN2* promoter [65, 66]. Because standard DHS analysis pipelines discard multi-mapped reads and the *HYDIN2* promoter sits in duplicated space, reads corresponding to DHS sites in fetal brain were remapped to a repeat-masked GRCh38 using mrsFAST-Ultra (version 3.3.11) [67] before being used to determine cut counts. Sample information including GEO accession numbers are shown in Additional file 1: Table S8.

### MIP exon sequencing

Human reference sequence (GRCh37) of coding exons from the ancestral paralog only (+/– 5 bp) was used as input to design single-molecule MIPs [68] using MIPgen [44]. Each MIP was designed to capture 112 bp of genomic sequence and included 40 bp unique to the target region (split between a ligation and an extension arm of the MIP), a universal 30 bp backbone, and a degenerate 8 bp molecular tag included on the extension arm. A total of 240 MIPs were designed to cover *HYDIN*. MIP phosphorylation, capture, and barcoding were performed as previously described [69]. Briefly, oligos were pooled together at equal concentrations (100 uM), phosphorylated, and an 800:1 excess of oligos was used for the genomic DNA capture (100 ng). Capture reactions were incubated at 60 °C for 18 h. Finished libraries were pooled together and sequenced using either MiSeq (2 × 150 bp) or HiSeq2000 (2 × 101 bp). Probe sequences can be found in Additional file 3: Table S9.

We used the MIPgen data analysis pipeline to map and filter reads in fastq format to a minimal human reference containing only the region containing the ancestral *HYDIN* paralog (chr16:70821397-71282326) included in our MIP design with the remainder of the genome, including *HYDIN2*, masked out. This masking ensured reads mapped to only the ancestral paralog for proper variant annotation. Discovery variant calling was performed across the entire ASD cohort per pooled sequence set containing up to 384 samples using FreeBayes (https://github.com/ekg/freebayes) with the following command: freebayes -b < sorted_bams > -f < masked_reference > -t < targeted_regions > -F 0.07 -C 2 -n 4. We removed any variants with the following feature: trinucleotide or homopolymer repeat, read depth ≤10, quality score ≤20, or with no alleles using previously described methods [70]. The resulting variant set was annotated using the Ensembl Variant Effect Predictor (VEP) [71] using the canonical transcript for each gene. Subsequently, for the ASD study, the complete list of coding variants was used to separately genotype cases and controls to assess overall frequency of events in each cohort: freebayes -b < sorted_bams > -f < masked_reference > -s < sample_list > -@ < variant_vcf > --only-use-input-alleles -F 0.07 -C 2 -n 4 --min-coverage 10.

### Tests for selection

The full-length ancestral *HYDIN* sequence (NM_001270974) was used as a BLAST query to obtain sequenced *HYDIN* transcripts from other primates. All codons from *HYDIN2* that could align with *HYDIN* were used. An MSA was generated using MAFFT and manually edited for obvious alignment errors. Bases aligning to the 15,366 nt ORF from human *HYDIN* were selected and a neighbor-joining tree was generated using MEGA. The alignment and tree were input into CODEML [72] and dN/dS values (omega) were estimated using the Nei-Gojobori method with pairwise deletion [73]. Branch-based tests were performed by allowing additional branches to vary in their dN/dS parameter and comparing the log-likelihood to the nested model. *p* values were calculated by performing a Chi-square test (df = 2) on twice the difference between the log-likelihood values for different models considered.

### *HYDIN* paralog-specific copy number genotyping using MIPs

*HYDIN* paralog-specific copy number was genotyped using a previously described method [36] with single-molecule MIPs [68]. Briefly, MIPs were designed to SUNs distinguishing *HYDIN* paralogs (Additional file 3: Table S10). MIP capture, library preparation, massively parallel sequencing, and data analysis allowed quantification of reads derived from each *HYDIN* paralog over each MIP target for each individual. These data were input to a program that output paralog-specific copy number calls and detected duplications, deletions, and interlocus gene conversion events. MIP data for each individual was visualized by plotting paralog-specific *HYDIN* copy number point estimates across the spatial extent of sequence shared between paralogs. These estimates were calculated at each MIP target by multiplying paralog-specific *HYDIN* read count relative frequencies by corresponding aggregate *HYDIN* copy number estimates called by the genotyping program. The algorithmic details of this program have been previously described [36]. In this case, the program considered 25 possible hidden underlying *HYDIN* paralog-specific copy number states, where both *HYDIN* and *HYDIN2* were allowed to possibly have copy numbers in the range of 0–4 (5 × 5 = 25 combinations). To enable detection of internal events, highest scoring paths through likelihood-based graphs allowing 0, 1, and 2 transitions between copy number states were considered, with the same biologically motivated restrictions on permitted transitions as previously detailed. Prior probabilities were set to reflect the observation that most humans have two copies of both *HYDIN* paralogs, with log-likelihoods of –15, –7.5, 0, –7.5, and –15 assigned to initial single-paralog copy number states of 0, 1, 2, 3, and 4, respectively. Probe sequences can be found in Additional file 3: Table S10.

### Array CGH

Array CGH was performed as previously described [13, 16] using a custom microarray (Agilent) with dense probe coverage across the chromosome 1q21 region.

## Additional files

Additional file 1: Figure S1.
*HYDIN* and associated chromosome 1 duplications, **Figure S2A.** Timing of first duplication of the 5' and 3' segments flanking *HYDIN2*, **Figure S2B.** Timing of second duplication of the 5' and 3' segments flanking *HYDIN2*, **Figure S3.** Paralog-specific copy number estimates for 236 individuals from the Human Genome Diversity Project (HGDP), **Figure S4.** *HYDIN* internal structural variation and interlocus gene conversion, **Figure S5.** 1q21 rearrangement breakpoint variability, **Figure S6.** The *HYDIN2* promoter corresponds to a peak of chromatin accessibility in fetal brain, **Table S1.** MIP-based copy-number genotyping of *HYDIN2*, **Table S2.** *HYDIN* duplication, deletion, and interlocus gene conversion events, **Table S3.** Locations of other copies of the *HYDIN2* promoter-associated duplication, their relationship to *NBPF*, and evidence for transcription, **Table S4.** Pairwise dN/dS values for *HYDIN* in primates, **Table S5.** Likely gene-disruptive events detected in *HYDIN*/*HYDIN2* by MIP-based sequencing of exons, **Table S6.** Phenotypes for patients having atypical chromosome 1q21 rearrangements, **Table S7.** Primers used in RACE and RT-PCR experiments, and **Table S8.** Fetal brain DNase I hypersensitivity samples with GEO accession numbers. (DOCX 5908 kb)
Additional file 2: Measured *HYDIN2* isoforms (.fasta). Fasta file of transcripts used for expression measurements. (FASTA 34 kb)
Additional file 3: Molecular inversion probe (MIP) sequences used for exon sequencing (**Table S9**) and copy number genotyping (**Table S10**). (XLSX 74 kb)
