# Supplementary material for: The birth of a human-specific neural gene by incomplete duplication and gene fusion
Source: Genome Biol. 2017 Mar 9;18:49. doi: 10.1186/s13059-017-1163-9 (PMC5345166; doi:10.1186/s13059-017-1163-9)
Supplement: Additional file 1: Figure S1. — HYDIN and associated chromosome 1 duplications, Figure S2A. Timing of first duplication of the 5' and 3' segments flanking HYDIN2, Figure S2B. Timing of second duplication of the 5' and 3' segments flanking HYDIN2, Figure S3. Paralog-specific copy number estimates for 236 individuals from the Human Genome Diversity Project (HGDP), Figure S4. HYDIN internal structural variation and interlocus gene conversion, Figure S5. 1q21 rearrangement breakpoint variability, Figure S6. The HYDIN2 promoter corresponds to a peak of chromatin accessibility in fetal brain, Table S1. MIP-based copy-number genotyping of HYDIN2, Table S2. HYDIN duplication, deletion, and interlocus gene conversion events, Table S3. Locations of other copies of the HYDIN2 promoter-associated duplication, their relationship to NBPF, and evidence for transcription, Table S4. Pairwise dN/dS values for HYDIN in primates, Table S5. Likely gene-disruptive events detected in HYDIN/HYDIN2 by MIP-based sequencing of exons, Table S6. Phenotypes for patients having atypical chromosome 1q21 rearrangements, Table S7. Primers used in RACE and RT-PCR experiments, and Table S8. Fetal brain DNase I hypersensitivity samples with GEO accession numbers. (DOCX 5908 kb) [file 13059_2017_1163_MOESM1_ESM.docx]

**ADDITIONAL FILE 1**


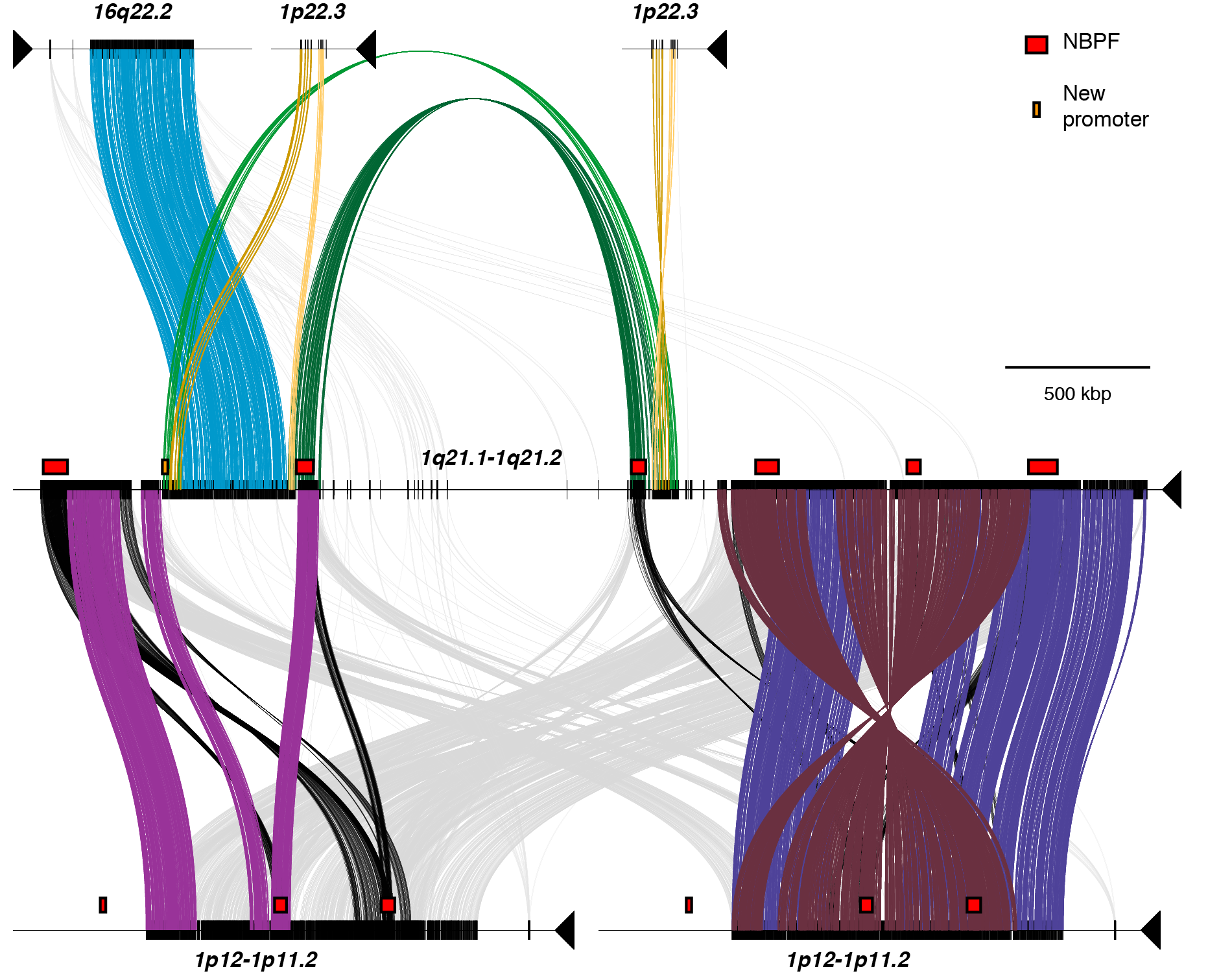


**Supplementary Figure 1. *HYDIN* and associated chromosome 1 duplications.** The segmental duplications that led to the formation of *HYDIN2* are seen in the context of larger genomic rearrangements on chromosome 1. Duplications are visualized using Miropeats (s = 800; Parsons, 1995). The central sequence represents chromosome 1q21.1-1q21.2 (chr1:146061572-150028860) with the *HYDIN* duplication in light blue. Also highlighted is the new *HYDIN2* promoter. The homology with chromosome 16q22.2 (chr16:70611384-71368670) and chromosome 1p22.3 (chr1:87315068-87609100) shown above the 1q21 region is the same as in Figure 1e. Shown below the 1q21 region is additional homology to chromosome 1p12-1p11.2 (chr1:119530046-121401465). This region is shown twice for clarity. Locations of the core duplicon gene *NBPF*, by GENCODE annotation, are highlighted as red boxes and are found at or near the breakpoints of most observed rearrangements, including the palindromic duplication shown at the bottom right (also described in O’Bleness, 2014).


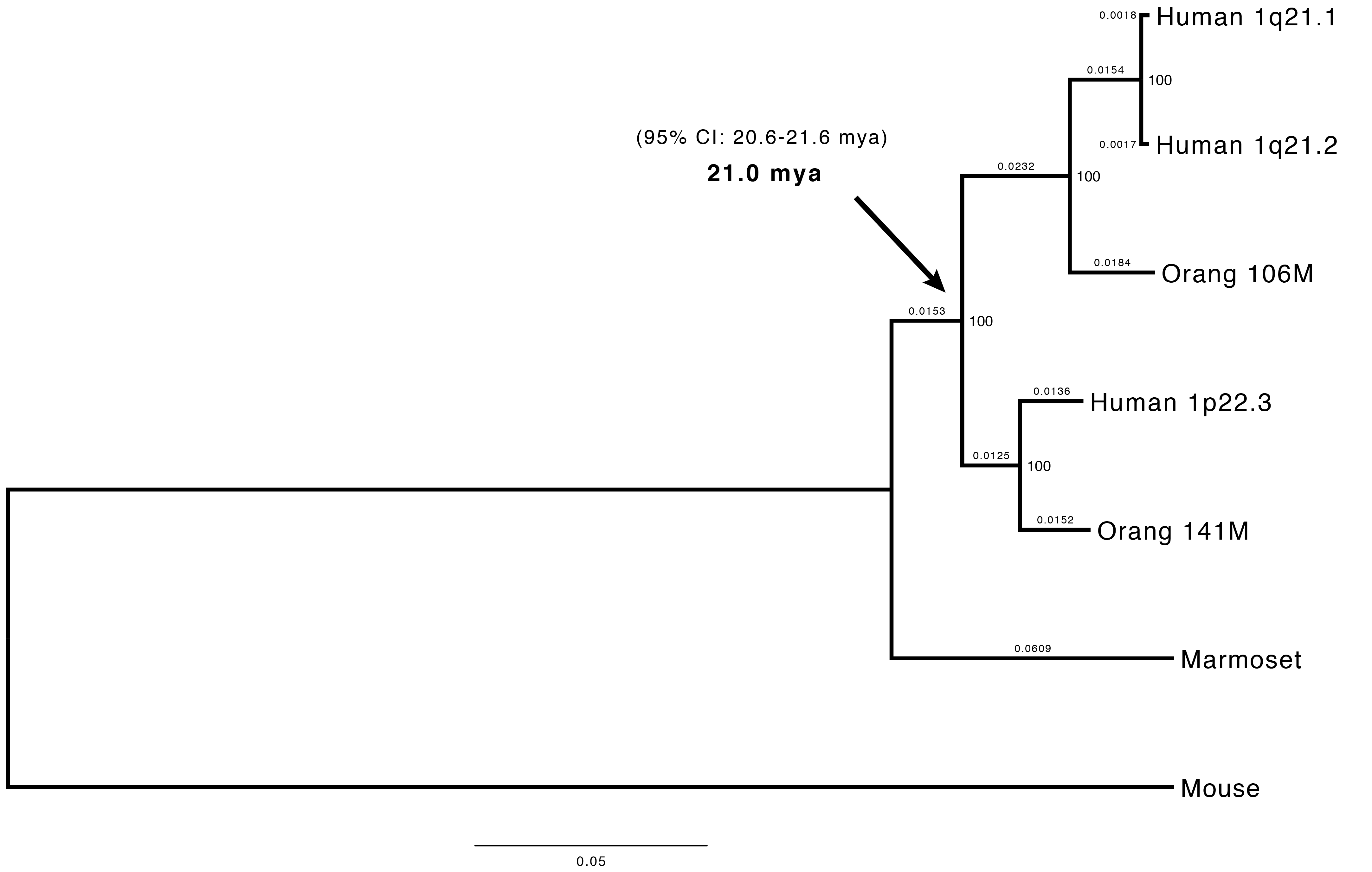


**Supplementary Figure 2A. Timing of first duplication of the 5**' **and 3**' **segments flanking *HYDIN2*.** We estimate that the segmental duplication (65 kbp) that was bisected by the *HYDIN2* duplication underwent an earlier duplication from chromosome 1p22 to chromosome 1q21 approximately 21.0 mya (95% CI: 20.6-21.6 mya). This is consistent with the observation that this segment is at haploid single-copy in marmoset, macaque, and baboon, and at haploid copy number 2 in gibbon and orangutan.

*Sequences*. Sequences homologous to the three human loci were identified in primates and in mouse using BLAT and the UCSC Genome Browser. A single homologous locus was identified in mouse, which was used to root the tree. One homologous locus was identified in marmoset, representing New World monkeys, and one homologous locus was identified in both the rhesus macaque and baboon, representing Old World monkeys. This suggests the duplication occurred after the divergence of apes from Old World monkeys. Within apes, two homologous loci were identified in gibbon and orangutan each, suggesting that the duplication occurred within the common ancestor of hominoids (lesser and greater apes). Multiple homologous loci of varying lengths were identified in chimpanzee, suggesting this region has undergone further rearrangement and amplification in that lineage.

*Alignment and tree building*. A 30,872 bp multiple sequence alignment (MSA) was generated using MAFFT with sequences deriving from the single locus in mouse and marmoset, the two loci in orangutan, and the three loci in human. The phylogenetic tree was inferred using the maximum-likelihood method with distances estimated using the Tamura-Nei model and tested with 50 bootstrap replicates. Branches are labeled with the number of substitutions per site and nodes are labeled with bootstrap support.

*Timing the duplication*. The two loci on human 1q21 pass the relative rate test (p = 0.65, outgroup orangutan), as do either with their ortholog in orangutan (p = 0.89 for 1q21.1 and p = 0.61 for 1q21.2, outgroup marmoset). The locus on human 1p22.3 and its closest orangutan ortholog also pass the relative rate test (p = 0.25). However, the 1q and 1p clades appear to be evolving at different rates (p < 0.00001 for both, outgroup marmoset), and neither clade passes the relative rate test with marmoset (p = 0.004 for 1q and p < 0.00001 for 1p, outgroup mouse). In marmoset, the derived sequence sits on the long arm of chromosome 7, consistent with known translocations that differentiate the species (Sherlock, 1996). All in all, we cannot assume the same local rate of neutral substitution between any pair in these three clades (marmoset, 1p clade, 1q clade).

We use the 1p22.3 clade to estimate the timing of the duplication since that region is syntenic to the original sequence in marmoset. These estimates are based on the proportion of genetic distance since divergence from marmoset that occurred since the duplication of our segment of interest.

*clade 1p*

D_human 1p22.3 branch_ = 0.013632

D_orangutan 141M branch_ = 0.015181

Average(D_orangutan 141M branch_, D_human 1p22.3 branch_) = 0.014407

D_1p until human-orang split_ = 0.012466

D_from divergence from marmoset to duplication_ = 0.015257

(Distance after duplication)/(Total distance since divergence from marmoset) = (0.014407 + 0.012466)/(0.014407 + 0.012466 + 0.015257) = 0.63785

Time of human–marmoset divergence (Glazko and Nei 2002) = 33 mya (range 32-36)

Estimate based on *clade 1p*

0.63785 * 33 mya = **21.0 mya** (95% CI: 20.6-21.6 mya)

We calculated 95% confidence intervals around our duplication timing estimate above using branch length error estimates and the following approach. First, for each branch in the tree, we set the branch length to a randomly chosen value between the actual branch length minus the branch length error (or zero if that value is negative) and the actual branch length plus the branch length error, inclusive. Second, we recomputed the timing estimate above using the same calculations as for the original tree except using the modified branch length values. For these calculations, we assumed a human–marmoset divergence of 33 mya. Third, we repeated the above two steps until we obtained one million modified trees and corresponding timing estimates. Finally, we sorted the estimates and reported the 25,000^th^ and 975,000^th^ sorted timing estimate values as the 95% confidence interval around the corresponding timing point estimate: 20.6-21.6 mya. Note that this error is less than the error that results from uncertainty in the timing estimate for human–marmoset divergence (Glazko and Nei, 2003).

This range of estimates, which places the duplication just after the divergence of apes and Old World monkeys (23 mya, range 21-25), is consistent with where we place the duplication in evolutionary history based on its presence and absence in reference genomes. It is important to note, however, that nonhuman primate reference genomes may be incomplete, especially in these duplicated regions.


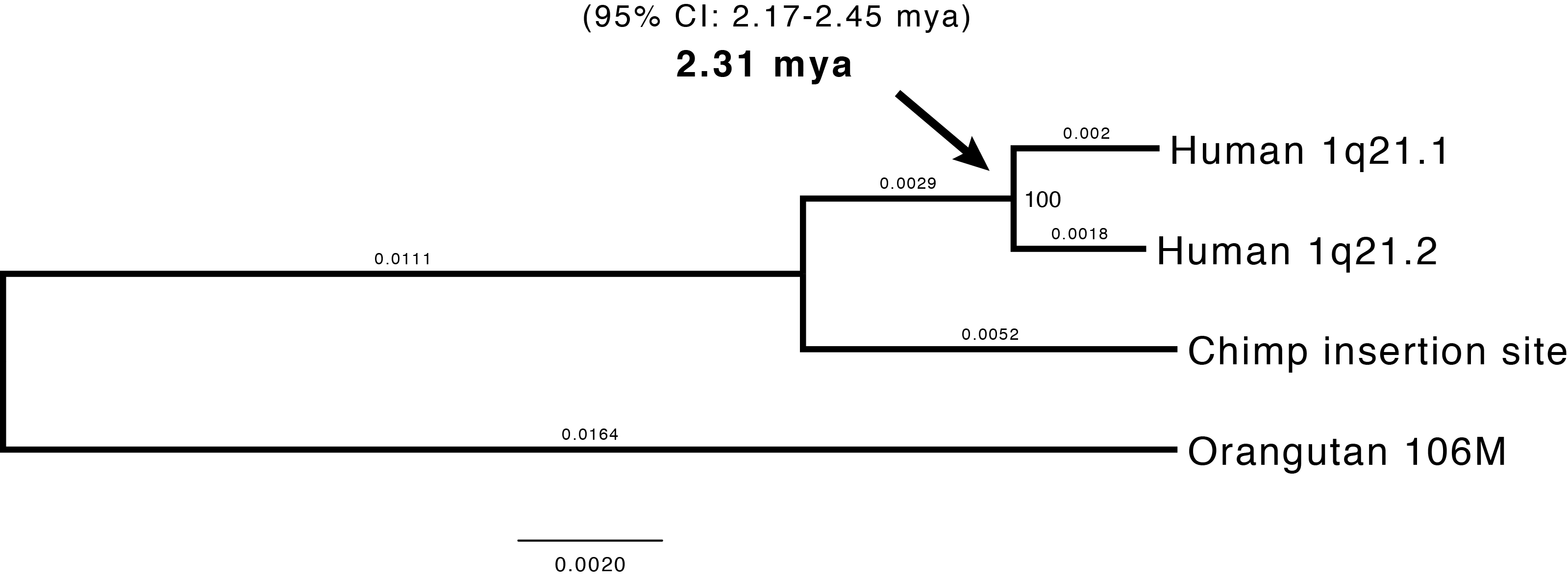


**Supplementary Figure 2B. Timing of second duplication of the 5**' **and 3**' **segments flanking *HYDIN2*.** To time the more recent duplication of the sequence immediately flanking *HYDIN2*, which occurred between 1q21.1 and 1q21.2, a new MSA was generated with sequence from the two human loci, the homologous chimpanzee locus, and the homologous orangutan locus. The sequences for the two human loci and the orangutan locus are the same as used in Figure S2a. Because this region appears to have undergone further duplication in chimpanzee (data not shown), we identified and sequenced a BAC containing homologous chimpanzee sequence (CH251-231E10). A 209,299 bp MSA was generated using MAFFT and manually edited for obvious alignment errors. The phylogenetic tree was inferred using the maximum-likelihood method with distances estimated using the Kimura 2-paramter model and tested with 50 bootstrap replicates. Branches are labeled with the number of substitutions per site and nodes are labeled with bootstrap support.

*Timing the human-specific duplication*. We similarly estimate the timing of the human-specific duplication of the segments that flank *HYDIN2* using orangutan as the outgroup, and a divergence time of 6 mya (Glazko and Nei, 2003), based on the proportion of genetic distance since divergence from chimpanzee that occurred since the duplication of our segment of interest. The human branches pass the relative rate test (p = 0.18).

D_average human 1q21.1/1q21.2 branch_ = (0.002032 + 0.001846)/2 = 0.001939

D_1q human until 1q21.1/1q21.2 split_ = 0.002932

D_1q human total_ = (0.002032+ 0.001846)/2 + 0.002932 = 0.004871

(Distance after duplication)/(Total evolutionary distance since divergence from chimpanzee) = 0.001939/(0.004871 + 0.005213) = 0.1923

0.1923 * 2 * 6 mya = **2.31 mya** (95% CI: 2.17-2.45 mya)*

This estimate is more recent than the estimate of the *HYDIN2* duplication, however it must necessarily have preceded the insertion of the duplicated *HYDIN2* sequence. We think it most likely that interlocus gene conversion is responsible for reducing the genetic divergence between these segments and distorting this timing estimate.


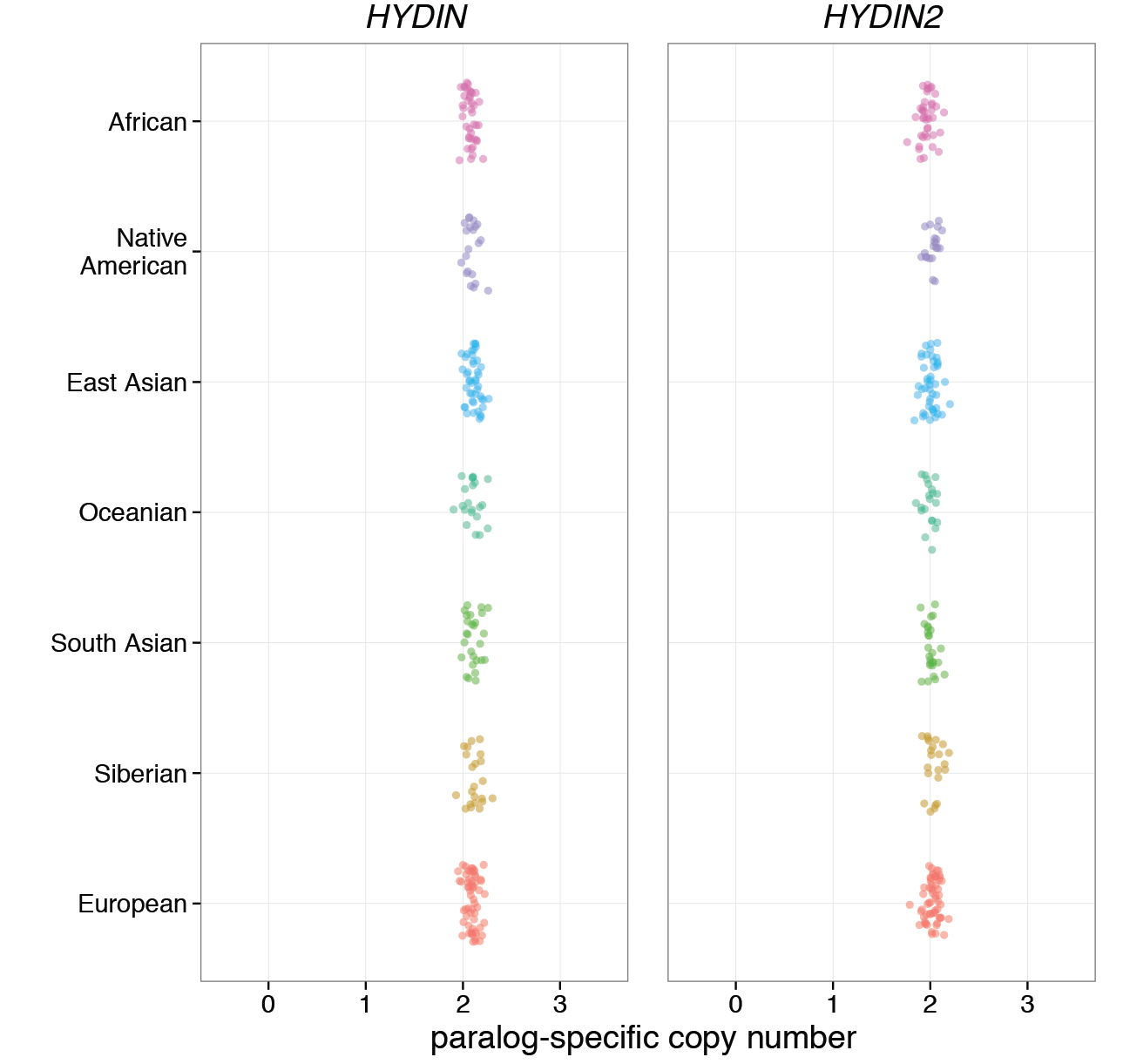


**Supplementary Figure 3. Paralog-specific copy number estimates for 236 individuals from the Human Genome Diversity Project (HGDP).** Whole-genome sequencing data from the HGDP were mapped to the *HYDIN* segmental duplication, and SUNK-based read depth was used to assess paralog-specific diploid copy number. Both paralogs are found at copy number 2 in all individuals shown, including 41 Africans, 21 Native Americans, 45 East Asians, 21 Oceanians, 27 South Asians, 22 Siberians, and 59 Europeans.


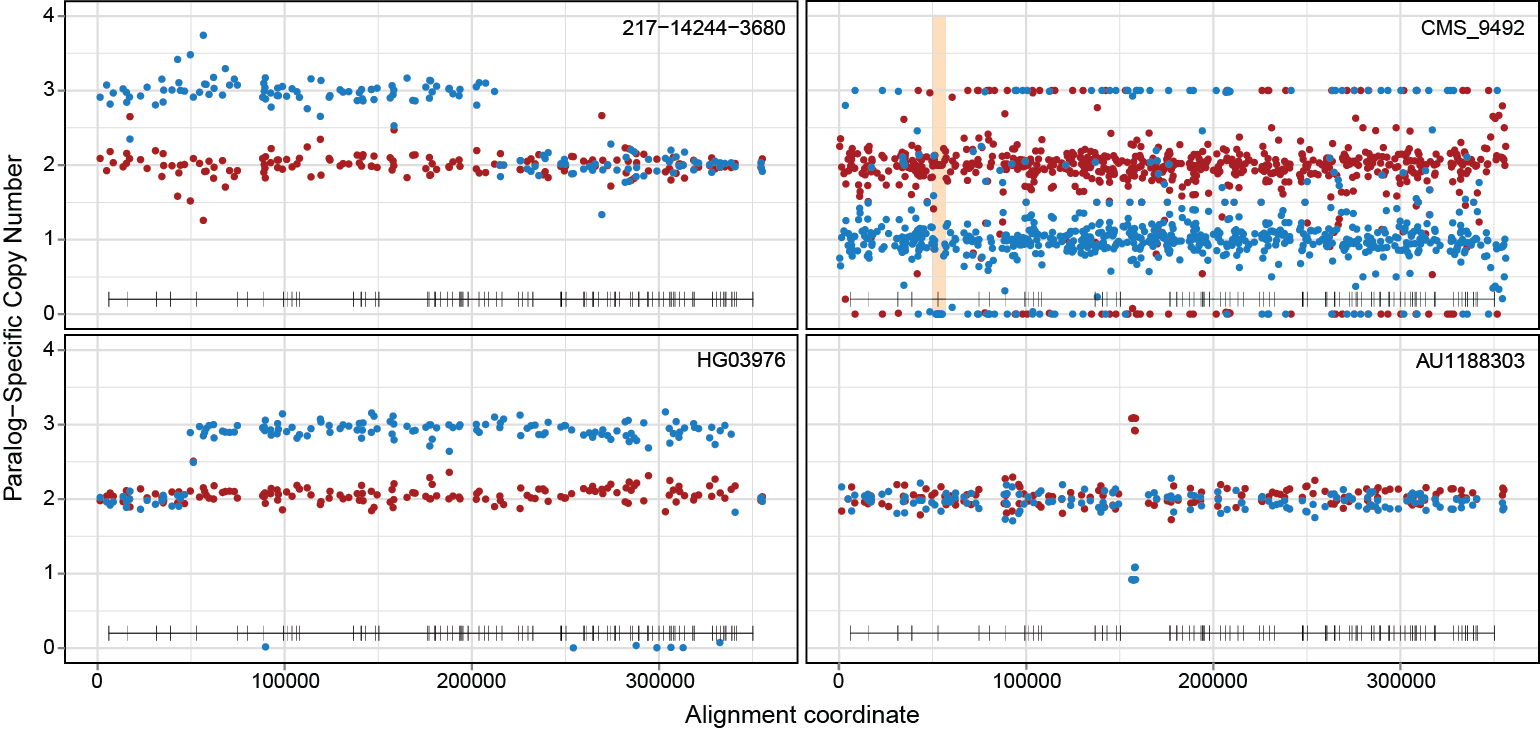


**Supplementary Figure 4. *HYDIN* internal structural variation and interlocus gene conversion.** Examples of structural variation within *HYDIN* paralogs and a putative interlocus gene conversion event. Each point shows a paralog-specific copy number estimate (red, *HYDIN*; blue, *HYDIN2*) based on sequencing data corresponding to a single MIP targeting sequence that distinguishes *HYDIN* paralogs. Sequencing reads were analyzed to compute paralog-specific read count relative frequencies for each MIP, which were multiplied by the aggregate estimated copy number at each target site to infer paralog-specific copy number. Shown are an ~212 kbp duplication affecting *HYDIN2* (upper left), an ~289 kbp duplication affecting *HYDIN2* (lower left), a putative ~3 kbp deletion affecting *HYDIN2* in a 1q21 microdeletion patient (orange highlight, upper right), and an ~2 kbp interlocus gene conversion event (lower right). Note that the putative *HYDIN2* deletion shown might instead reflect interlocus gene conversion—all reads for 11 consecutive MIPs over the highlighted interval mapped to *HYDIN*, consistent with zero copies of *HYDIN2* and either two or three copies of *HYDIN*. Also note that the interlocus gene conversion event identified in AU1188303 is the same as that discovered in 11094.s1 (**Figure 2C**, bottom right), indicating this event likely segregates at very low frequency. 153 MIPs were used for genotyping all individuals shown except CMS_9492, a 1q21 microdeletion patient genotyped with 717 MIPs. All events shown were detected by an automated caller. Duplicated exons based on the canonical *HYDIN* gene model are indicated at the bottom of each plot.


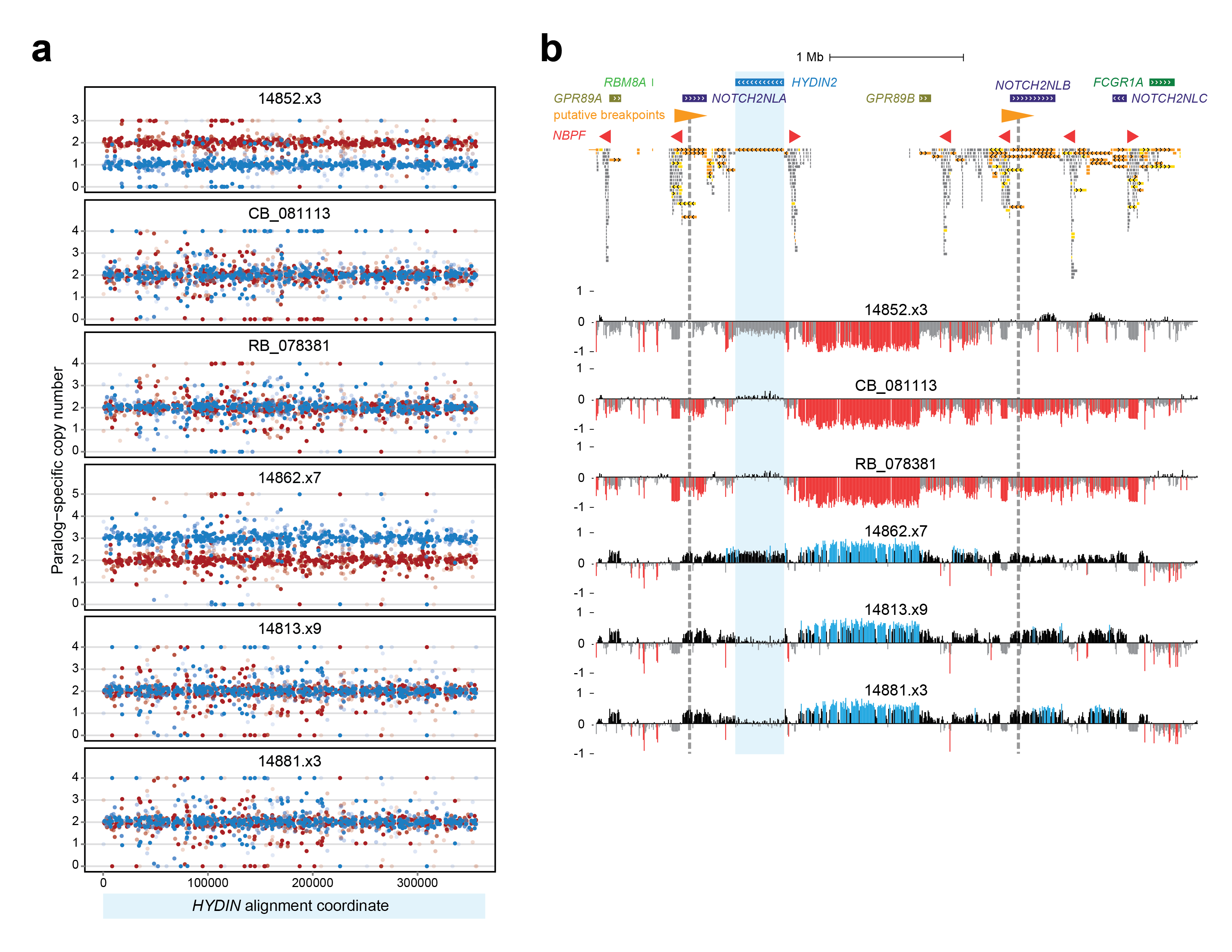


**Supplementary Figure 5. 1q21 rearrangement breakpoint variability. a)** 717 MIPs targeting regions that distinguish *HYDIN* paralogs were employed to genotype *HYDIN* paralog-specific copy number in 48 1q21 microdeletion and 25 1q21 microduplication patients. Points show *HYDIN* paralog-specific copy number estimates (red, *HYDIN*; blue, *HYDIN2*) for three microdeletion patients (14852.x3, CB_081113, and RB_078381) and three microduplication patients (14862.x7, 14813.x9, and 14881.x3). These estimates were calculated as the product of the paralog-specific read count relative frequency for a particular MIP and the aggregate estimated copy number at the corresponding target site. The MIP results indicate that 1q21 rearrangements do not always include *HYDIN2*. **b)** The segmental duplication organization of a 4.5 Mbp region at chromosome 1q21 (GRCh38 chr1:145,500,001-150,000,000) is shown along with array CGH profiles for the individuals in panel a. Thin colored boxes indicate sequences duplicated between this region and another genomic locus, with colors corresponding to sequence identity (orange = 99% or above, yellow = 98%–99%, gray = 90%–98%) and markings showing orientation (right-pointing, directly oriented; left-pointing, inversely oriented) between duplication pairs. Thick colored boxes highlight locations of several duplicated genes. Orange triangles indicate high-identity, directly oriented *NOTCH2NL-NBPF* duplications, with putative breakpoints of the canonical 1q21 rearrangement shown as vertical gray dashed lines. Shown below are the locations of *NBPF* core duplicons. Array CGH confirms 1q21 rearrangements in these individuals, and array data over *HYDIN2* (blue highlight) indicate a loss or gain in some (14852.x3 and 14862.x7) but not others (all other individuals shown), validating the MIP data shown in panel a.

**
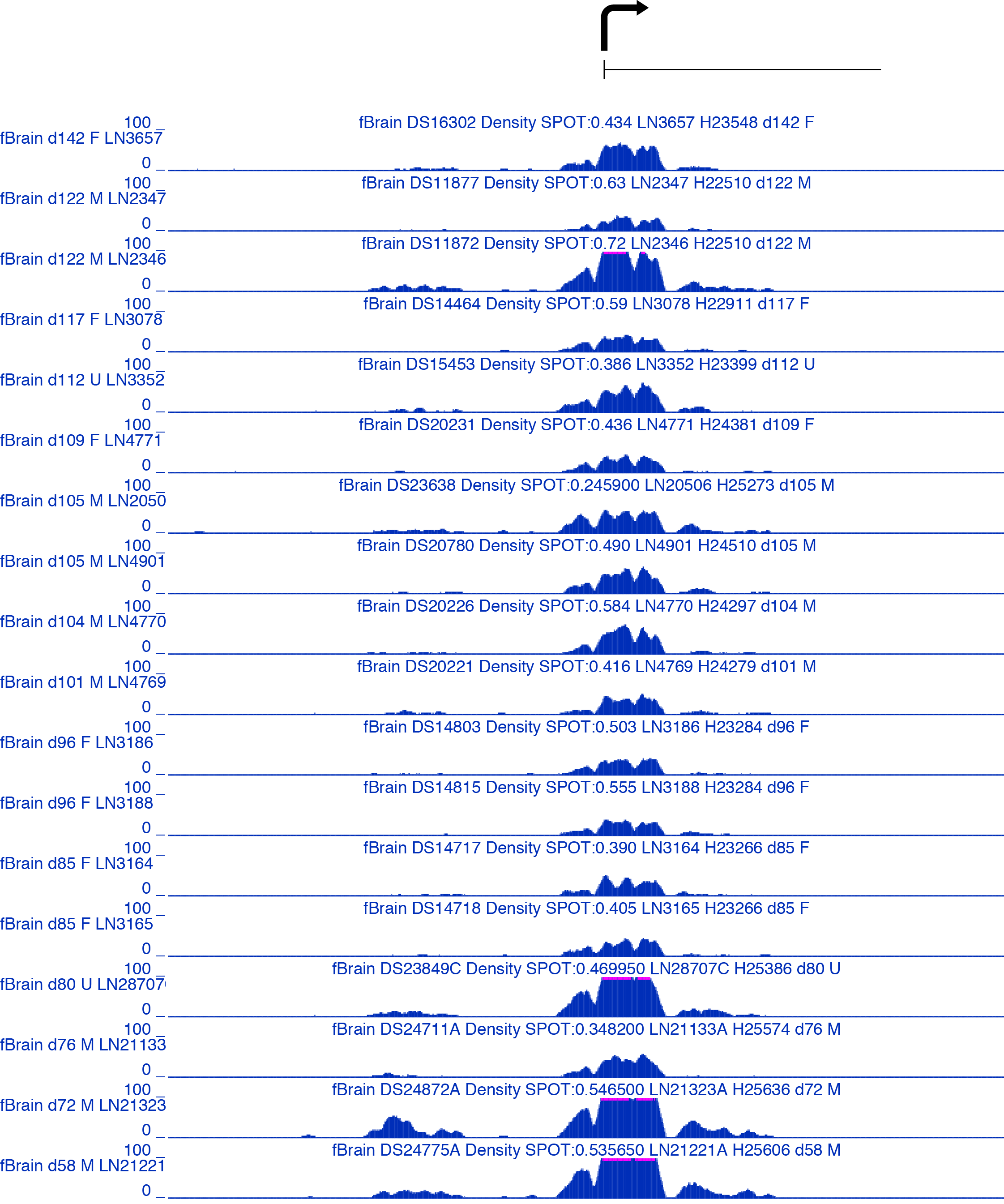
**

**Supplementary Figure 6. The *HYDIN2* promoter corresponds to a peak of chromatin accessibility in fetal brain.** Reads indicating sites of chromatin accessibility as determined by sensitivity to DNase I digestion from various fetal brain time points (day 58 – day 142) were mapped using mrsFAST-Ultra (Hach, 2014) to allow for measurement over duplicate sequence. Shown is a ~14 kbp region (chr1:146479496-146493419) surrounding the acquired promoter and first exon of *HYDIN2*, with a peak is visible at all time points. See Table S8 for sample information.


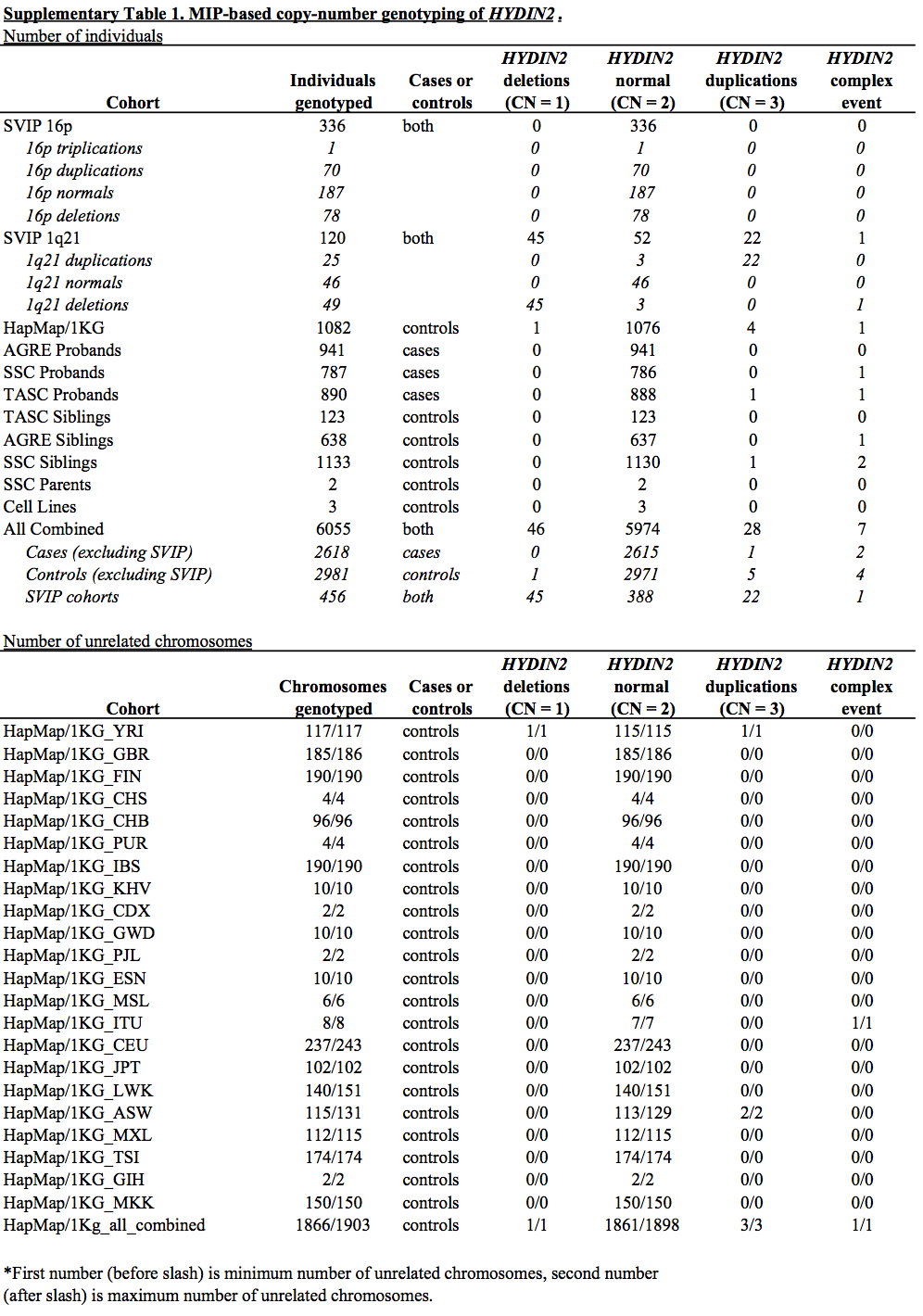


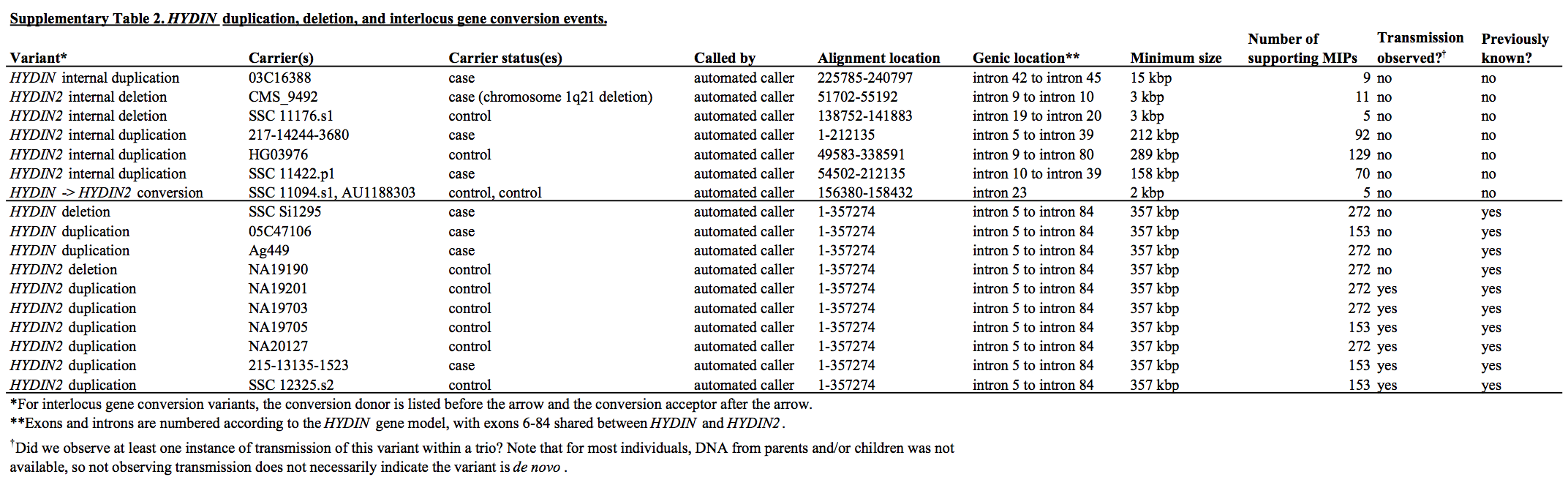


**
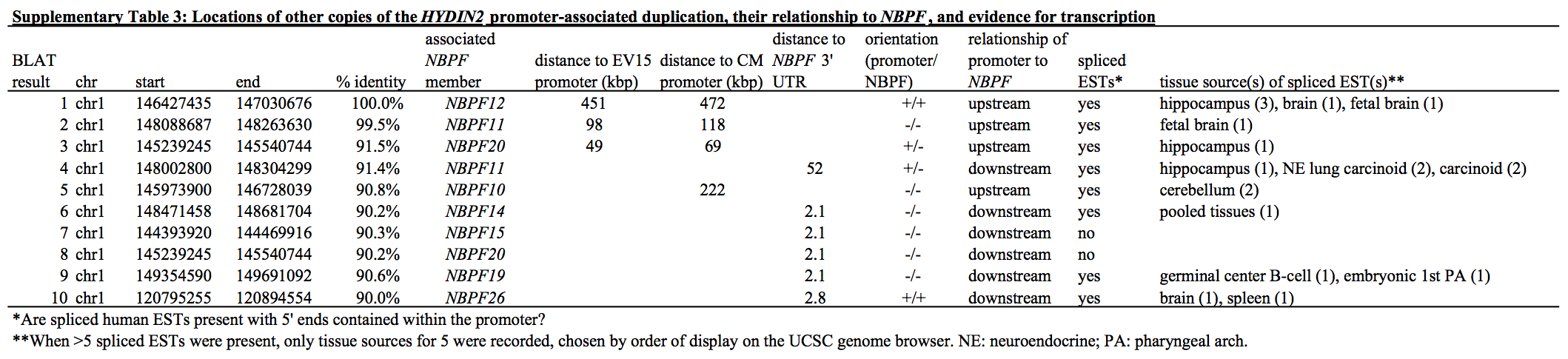
**


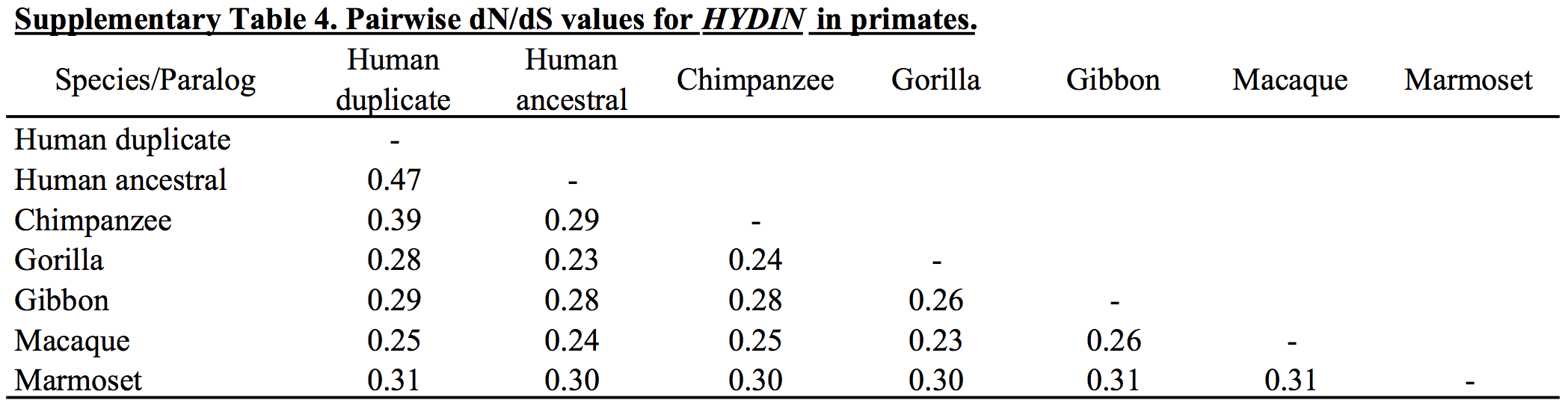


**
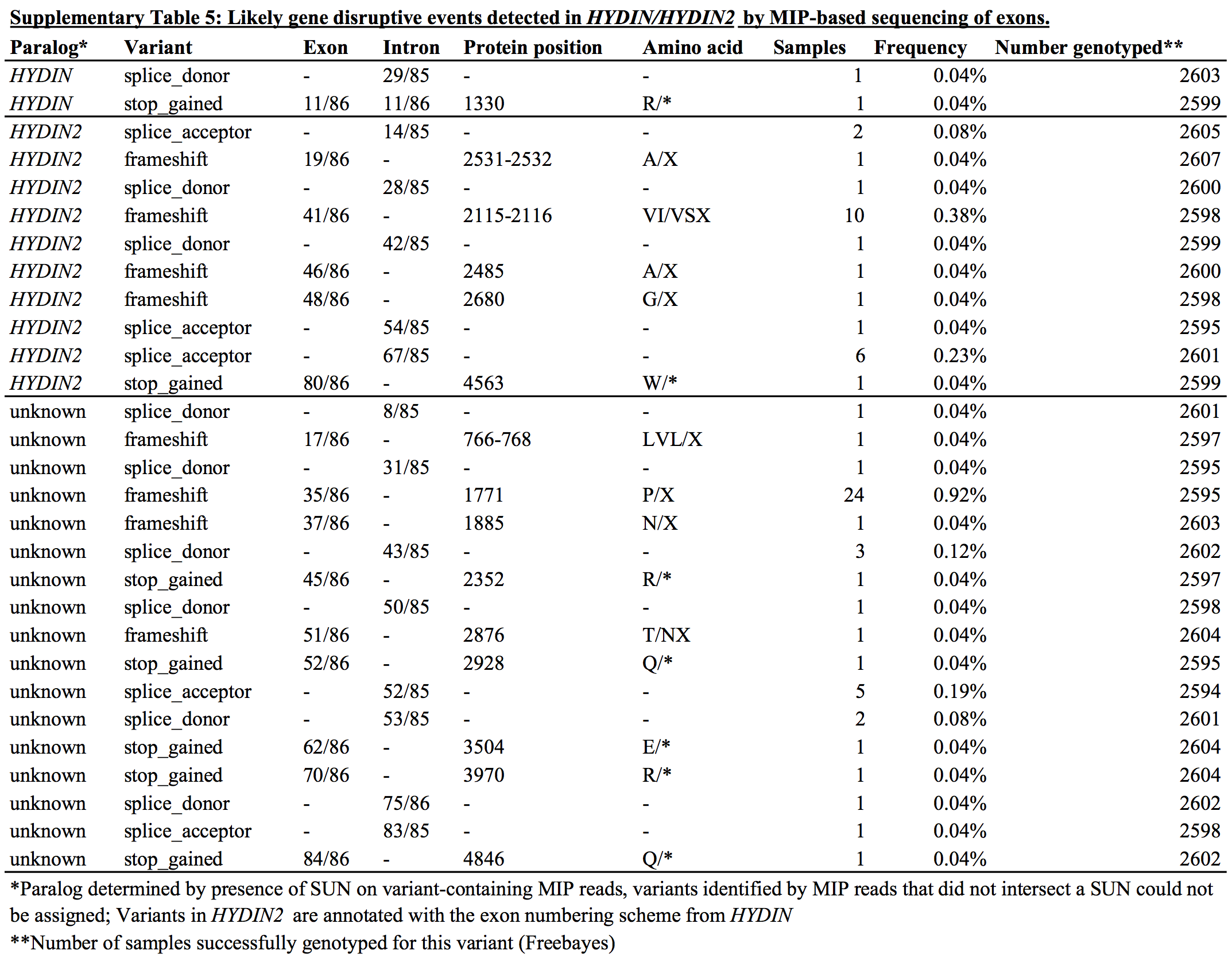
**


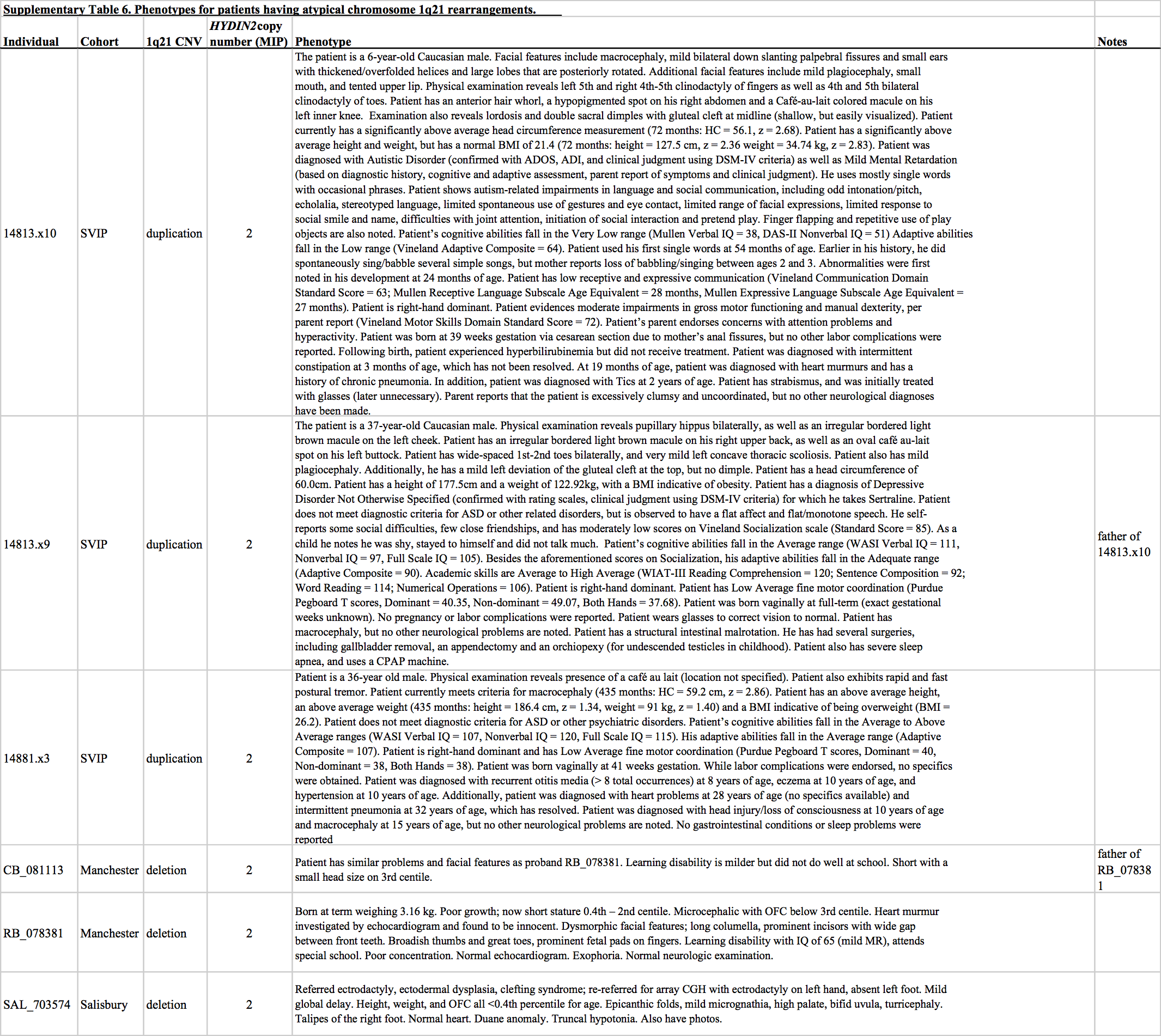


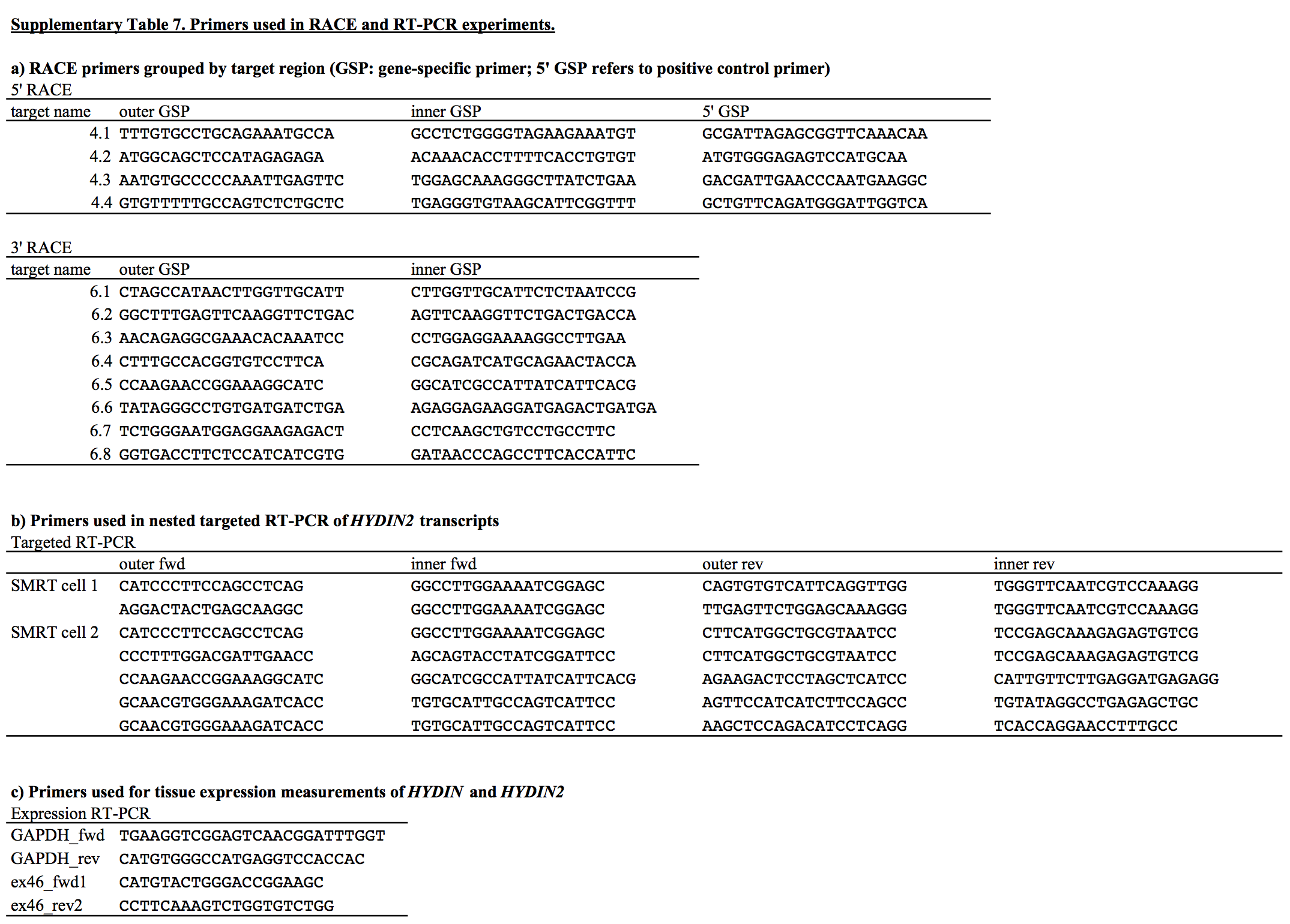


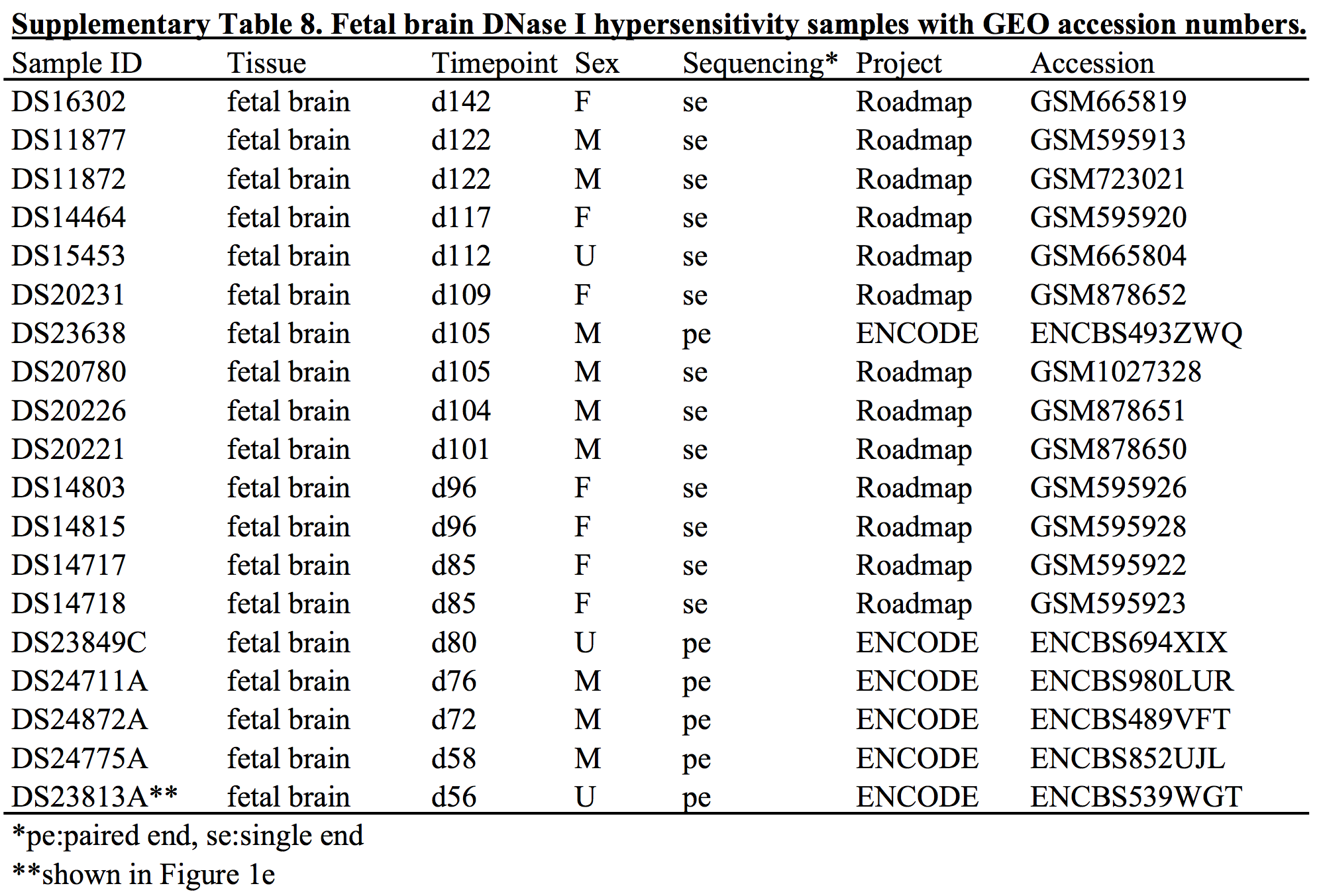


**Supplementary Tables 9 and 10, which contain a list of MIP sequences used, can be found in additional file 3.**
